# Liquid biopsies for the monitoring of gliomas and brain metastases in adults

**DOI:** 10.1007/s00401-025-02880-9

**Published:** 2025-04-26

**Authors:** Govert Dwarshuis, Lente L. Kroon, Dieta Brandsma, David P. Noske, Myron G. Best, Nik Sol

**Affiliations:** 1 Department of Neurosurgery, Brain Tumor Center Amsterdam, Cancer Center Amsterdam, Amsterdam UMC, Amsterdam, The Netherlands; 2 Department of Neurology, Netherlands Cancer Institute-Antoni Van Leeuwenhoek, Amsterdam, The Netherlands

**Keywords:** Liquid biopsy, Biomarkers, Monitoring, Brain tumors, Glioma, Brain metastases

## Abstract

Clinical evaluation and MR imaging are currently the cornerstone of brain tumor progression monitoring. However, this is complicated by the occurrence of treatment effects such as pseudoprogression and radionecrosis. While essential for patient management, the distinction from true progression remains a significant challenge. Moreover, MR imaging provides limited real-time insights into tumor heterogeneity, genetic divergence, and treatment resistance. Although surgical histopathological biopsies can yield additional valuable information, they are not always conclusive, invasive, and therefore, not suitable for longitudinal measurements. In the era of precision medicine, there is a critical need for minimally invasive, accurate, and cost-effective monitoring methods for both primary brain tumors and brain metastases. Liquid biopsies have emerged as a potential candidate. Various analytes, including circulating nucleic acids, extracellular vesicles, platelet RNAs, and circulating tumor cells, can be obtained from whole blood and its derivatives, as well as other body fluids such as cerebrospinal fluid. In this narrative review, we outline the potential of liquid biopsies for the management of gliomas and brain metastases in adults and emphasize their utility in monitoring disease progression and treatment response. We discuss the most studied biofluids and analytes, along with their respective advantages and downsides. Furthermore, we address key considerations for future research and biobanking to pave the way for clinical implementation.

## Introduction

Among primary brain tumors, gliomas are the most common with 6.5 per 100,000 individuals diagnosed annually in the United States [96]. The prognosis varies widely depending on glioma subtype and grade, ranging from a few months to several decades, with each subtype requiring different treatment strategies [81]. Brain metastases are the most prevalent brain tumors, affecting 10–40% of patients with solid cancers, primarily those with lung (19–40%) or breast cancer (6–22%), or melanoma (6–15%) [13, 18, 62, 93]. The improvements in systemic disease control and better imaging technologies have led to a significant increase of brain metastases [62]. Brain metastasis diagnosis frequently relies on clinical information and imaging combined with prior histopathological information from the primary tumor or other metastases. However, increasing knowledge of (molecular) heterogeneity and resistance mechanisms in brain metastasis as compared to primary tumor and systemic metastases has emphasized the limitations of relying solely on prior histopathology of the primary tumor, particularly in the context of targeted therapies [84]. Accurate and up-to-date molecular information of the primary tumor and its metastases, is therefore, essential to guide and monitor treatment.

The current monitoring strategies for both primary and secondary brain tumors rely heavily on magnetic resonance (MR) imaging, supplemented by clinical evaluation and, if indicated, tissue histopathological analysis. The higher-grade gliomas warrant more frequent imaging, typically every 3–6 months, burdening patients, scanner capacity, and healthcare funds [81]. Imaging alone does not provide information on the biology underpinning tumor response and treatment resistance. Notoriously challenging is the increase of contrast-enhancing lesions on MR imaging following (chemo-)radiation. This can be due to true tumor progression or may be treatment-induced, such as pseudoprogression or radionecrosis. These phenomena occur in approximately 30% of glioblastoma patients and in up to 30% of patients with brain metastasis [3, 134]. These treatment-induced imaging effects, relevant in both glioma and brain metastasis care, complicate clinical decision making as they may be misinterpreted as tumor progression [125, 131]. Advanced MR imaging modalities, such as spectroscopy, perfusion, and diffusion-weighted imaging may aid the discrimination from true tumor progression but are far from perfect [34, 77]. Needle biopsy or even repeat resection and histopathological analysis may not yield a conclusive answer, partially debit to tumor heterogeneity, and the lack of pathological definition of radionecrosis versus tumor progression. These factors emphasize the need for additional minimally invasive, accurate and affordable methods for the monitoring of brain tumors.

Over the recent decades, liquid biopsies have emerged as a promising candidate, including circulating nucleic acids, extracellular vesicles, platelet RNAs, and circulating tumor cells. These can be collected from whole blood and its derivates, but also from urine and cerebrospinal fluid (CSF). In the current era of personalized medicine, which has significantly improved the survival of cancer patients, increasing attention is being directed toward the use of liquid biopsies for disease monitoring. A decade ago, the potential of liquid biopsies has been extensively discussed in this journal, viz. Best et al. [22], which primarily focused on diagnostic capabilities of blood biomarkers for glioma diagnostics. As treatment monitoring appears to be the most relevant application of liquid biopsies in a neuro-oncological setting, we here present an initial overview of the current liquid biopsy platforms available for monitoring gliomas and brain metastases in adults. The value of liquid biopsies for molecular diagnosis and monitoring treatment response in pediatric brain tumors has been described previously [44, 124]. We provide perspective on the use of liquid biopsies for brain tumor monitoring and considerations for effective biobanking in clinical studies. The studies from this literature search are discussed per biomolecule for both diffuse gliomas and brain metastases. We discuss monitoring markers for diffuse glioma patients, which mainly focus on the distinction of tumor progression from pseudoprogression. In contrast, brain metastasis monitoring is primarily focused on differentiating radionecrosis from intracranial tumor progression. As a first step towards achieving this, a large number of studies have identified markers that differentiate between systemic and intracranial disease.

We expect liquid biopsies to complement histopathological, molecular and imaging diagnostics in the multidisciplinary treatment of neuro-oncological patients.

## Circulating nucleic acids

Circulating nucleic acids encompass cell-free DNA (cfDNA) and RNA. CfDNA has a half-life of < 1.5 h, theoretically allowing for real-time depiction of tumor activity [127]. Circulating tumor DNA (ctDNA) specifically refers to the tumor-derived subset of cfDNA, which is mainly shed from tumor cells during necrosis and apoptosis. In blood, ctDNA typically constitutes < 1% of total cfDNA [32, 50]. Since it is not always possible to differentiate between cfDNA derived from normal cells and ctDNA, we will from here on use the term ‘cfDNA’.

For over a decade, cfDNA and RNA have been investigated as potential cancer biomarkers [36]. This was primarily initiated by the seminal paper of Bettegowda et al. (2014), describing the detection of tumor-derived DNA molecules in blood from 15 different tumor types, with detection rates increasing as tumor stages advance [23]. Potential purposes range from diagnosis and identification of treatment targets to monitoring for minimal residual disease [83]. As tumor-derived cfDNA detection in blood has been difficult in central nervous system (CNS) tumors, it has been suggested that the blood–brain barrier (BBB) hampers release of cfDNA in the circulation. In brain metastases, a low cerebral tumor load compared to systemic metastases may also contribute to this reduced detection efficiency.

### Circulating nucleic acids in glioma

#### DNA

Starting with detection rates below 10% in 2014, the detectability of glioma cfDNA in blood plasma has improved over time [23]. More recent studies have detected glioblastoma somatic alterations in cfDNA in up to 83% of patients [10, 24, 37, 86, 89, 99, 114, 143]. Novel methods to detect cfDNA keep emerging rapidly, such as individual tumor-guided sequencing. Using an assay based on each patient’s individual tumor characteristics, Moulière et al. detected tumor-derived cfDNA in a cohort of IDH-wild type glioblastoma patients in CSF of 7/8 (88%) patients, in plasma of 10/12 (83%) patients, and in urine of 6/8 (75%) patients [86]. Tumor-derived cfDNA was more fragmented than non-mutant DNA in these biofluids. Selection of specifically fragmented DNA molecules may thus enhance detection sensitivity, as well as the presence of somatic mutations. Muralidharan et al. showed that in TERT-promoter mutant glioma (81% IDH-wildtype), this mutation can be detected in 63% of cases in plasma cfDNA employing digital droplet PCR (ddPCR) [89]. Moreover, in longitudinal monitoring of five patients, the TERT mutant allele frequency (MAF), i.e., the proportion of tumor-derived DNA among total detected cfDNA, decreased following resection and chemoradiation and increased upon tumor progression. Although TERT MAF did not correlate with MRI tumor volume, contrast-enhancing tumors had increased TERT MAF-values, indicating more tumor DNA leakage from a more disrupted and thereby more permeable BBB. Although not directly applicable to non-mutant TERT promoter gliomas (i.e., 40% of gliomas), this study delivers an important proof-of-concept regarding cfDNA as a blood-based monitoring marker in glioma [60]. In blood plasma of eight patients with glioblastoma, Iorgulescu et al. detected tumor cfDNA with 88% sensitivity and 99% specificity using their proprietary MAESTRO-Pool (minor allele enriched sequencing through recognition oligonucleotides) assay. Additionally, they suggest that it may help distinguish true progression from pseudoprogression as defined by follow-up MR imaging and histopathology, although longitudinal samples were collected in only seven patients [48]. Furthermore, two pilot studies noted that total cfDNA concentration in plasma might provide prognostic and monitoring value in glioblastoma regardless of whether somatic alterations were detected [10, 95]. Lower-grade gliomas are more difficult to detect as MAF decreases with glioma grade [99].

#### Epigenetics

CfDNA analysis also encompasses epigenetic alterations, such as methylation patterns. In patients with glioma, Nassiri et al. analyzed plasma-based DNA methylation profiles with cell-free methylated DNA immunoprecipitation and high-throughput sequencing (cfMeDIP-seq) and employed machine learning to distinguish gliomas from other brain tumor types, extracranial cancer, and healthy individuals with high accuracy (AUC 0.99; 95% confidence interval (CI): 0.96–1.00) [92]. The detection of specific types of glioma within a subgroup of common intracranial tumors was more challenging (IDH-wild type versus others: AUC 0.71; 95% CI 0.53–0.90; IDH-mutant versus others: AUC 0.82; 95% CI 0.66–0.98). Sabedot et al. developed a serum-based glioma-epigenetic liquid biopsy (GeLB) score that allowed for discrimination between 149 glioma patients and patients with various other brain tumor types with 98% accuracy [112]. Additionally, they were able to distinguish tumor progression from pseudoprogression in two patients with astrocytoma grade 2 and one patient with glioblastoma.

#### RNA

RNA is increasingly studied as a liquid biomarker source. Although unbound mRNA is unstable in blood, RNA that forms protein complexes, such as microRNA (miRNA), are more resistant to RNAses [9, 104]. Other RNA, such as long non-coding RNA and circular RNA (circRNA), are intrinsically more resistant to degradation and thus detectable in blood [46, 128].

MiRNA consists of small, non-coding fragments of RNA that serve as protein expression regulators. Distinct tissue miRNA profiles have been found in various cancer types, including brain tumors [72]. In contrast to cfDNA, miRNA seems to be less dependent on BBB disruption to enter the circulation or is released by ‘bystander’ cells (e.g., endothelial or immune cells) [85].

One study screened the expression of 739 miRNAs in serum obtained from patients with diffuse astrocytoma, anaplastic astrocytoma, or glioblastoma (according to the 2007 WHO classification) and age- and sex-matched healthy controls and found a panel of 108 differentially expressed miRNAs [142]. After subsequent validation in 90 astrocytoma patients and 110 healthy controls, the resulting 9-miRNA panel reached an AUC of 0.97 for the identification of astrocytoma cases, with a significant decrease of these miRNAs following tumor surgery in 73 patients. Another study aimed to identify a miRNA serum biomarker to monitor lower-grade glioma and glioblastoma patients post-treatment [85]. This group also first identified a 9-miRNA panel, demonstrating near-perfect accuracy (AUC 0.998) distinguishing between glioma patients and healthy controls. The authors analyzed 11 patients with longitudinal blood samples and found a close correlation between miR-223 and lower-grade glioma volume, and miR-320e and glioblastoma volume as measured on MR imaging. In two cases of pseudoprogression, miRNA levels did not increase. These studies suggest the potential of miRNA as a biomarker in glioma monitoring and warrant a prospective validation study.

In ﻿summary, circulating nucleic acids show promise in distinguishing between glioma subtypes, differentiating gliomas from other brain tumors, and may allow treatment monitoring. However, many studies have small sample sizes, are retrospective, and lack external validation. Furthermore, the detection of glioma-derived cfDNA in blood is challenging due to its low abundance and short half-life. Advancements in detection techniques as well as multimodal testing approaches could improve glioma monitoring. As miRNA is more stable than cfDNA and easier to detect in the blood, they show promise in glioma detection. Clues towards their monitoring potential have been found but require further investigation. 

### Circulating nucleic acids in brain metastasis

#### DNA

Brain metastasis-derived circulating nucleic acids are difficult to identify and to distinguish from those originating from systemic metastases. Several studies have explored the role of cfDNA for diagnosis and monitoring of brain metastasis in patients with metastatic solid tumors. Liang et al. detected cfDNA in blood of 28% (2/7) of cancer patients with brain metastases, with genetic alterations matching those in brain tumor tissue [67]. However, it was not specified whether these patients also had systemic metastases, which may have positively affected cfDNA detectability in the blood.

Several studies have shown that cfDNA detection is particularly difficult in isolated progressive intracranial disease. First, Aldea et al. identified plasma cfDNA in 52% (*n* = 28/54) of NSCLC patients with intracranial progression only (iCNS), compared to 84% (*n* = 83/99) with extracranial (eCNS) and 92% (*n* = 86/94) with concurrent extra- and intracranial progression (cCNS) [5]. Driver and resistance genetic alterations were also lower in the iCNS group compared to the eCNS and cCNS group, respectively 37% versus 77% and 73% for driver alterations and 6% versus 45% and 44% for resistance alterations. Second, Alder et al. assessed genomic alterations in serum cfDNA of 253 patients with brain metastases of various solid cancers, primarily breast cancer (12%) and NSCLC (76.4%) [6]. The proportion of detected MAFs ≥ 1% was 65.5%, 80.6%, and 73.4% (*p* = 0.40) for the iCNS (*n* = 29), cCNS (*n* = 160), and eCNS (*n* = 64) group, respectively. Additionally, the median MAF percentage per patient identified in any gene was also higher in the cCNS group (median 4.75) compared to the iCNS (median 1.6) and eCNS group (median 2.55, *p* = 0.003). Although the identified MAFs were lower in the iCNS patients, the researchers successfully identified unique genomic alternations in their cfDNA. Furthermore, they demonstrated a comparable ability to detect actionable mutations in cfDNA of patients with iCNS and those with extracranial progression. These findings suggest that cfDNA might find an application in characterizing molecular profiles of brain-metastases, thereby informing and optimizing treatment strategies. Kim et al. showed plasma cfDNA’s clinical utility. Among 164 patients with positive tissue or plasma EGFR mutation tests, 34 (20.7%) were detected only in plasma, leading to first-line EGFR TKI treatment in 85.3% (29/34) of patients [54]. Furthermore, they found a significant association between EGFR mutation status in plasma cfDNA and the presence of brain metastasis in 311 treatment-naive stage IV non-small cell lung cancer (NSCLC) patients, with an adjusted odds ratio of 2.73 (95% CI 1.39–5.36; *p* = 0.003).

Two additional studies analyzed plasma cfDNA in (brain) metastatic melanoma patients before and during anti-PD1 therapy. Seremet et al. detected BRAF^V600E/K^ mutations in 47% (22/47) and NRAS^Q61/G12/G13^ mutations in 38% (6/16) baseline samples but found no detectable cfDNA in 36 samples collected at baseline and subsequent assessment during therapy from ten patients with progressive intracranial disease only [115]. Lee et al. detected tumor-derived cfDNA in plasma of 53% (40/76) patients with active brain metastasis at baseline and in 24% during therapy [64]. Both studies linked undetectable cfDNA levels at baseline and follow-up with better survival. However, both studies failed to detect cfDNA in patients with isolated brain metastases.

In conclusion, previous studies illustrate the challenges of detecting isolated progressive brain metastases and monitoring brain metastasis response to therapy using cfDNA analysis in blood. Despite these difficulties, the unique genomic alterations can still be identified in cfDNA from patients with isolated intracerebral progression, offering potential insights for treatment decisions.

#### Epigenetics

Methylation patterns could also aid in early diagnosis in patients at risk for brain metastases and help identify an unknown primary tumor. Barciszewska found that differences in 5-methylcytocine (m^5^C) content between DNA of matched brain metastasis tissue and peripheral blood samples were significantly associated with primary tumor type and negatively correlated with histopathological tumor grade (from G1: highly differentiated, least malignant to G3: low differentiated, most malignant) [12]. Furthermore, m^5^C content in DNA of matched brain metastasis tissue and peripheral blood samples was strongly correlated. Pangeni et al. analyzed cfDNA in plasma from brain metastatic breast cancer patients and found concordant methylation patterns in cfDNA and brain metastasis tissue [97]. Zuccato et al. evaluated DNA methylation patterns of lung cancer patients with and without brain metastasis [144]. Plasma methylome-based cfMeDIP-seq enabled them to identify brain metastasis in patients with lung cancer with high accuracy (AUC = 0.80, 95%-CI 0.68–0.93). Additionally, the plasma methylome signals of patients with brain metastasis correlated well with matched brain tumor tissue methylation values. Thus, the methylation patterns in plasma may harbor potential as a diagnostic and prognostic tool for brain metastasis.

In general, it is known that cancer inflicts damage to the surrounding organ tissue. Lubotzky et al. demonstrated that cancer-induced cell death in organs is reflected in tissue-specific cfDNA methylation patterns [73]. Although levels of brain-derived cfDNA in plasma were low compared to cfDNA derived from other organs, they were measurable in most patients with brain metastases (neuron-derived in 27/29 patients, oligodendrocyte-derived in 25/29 patients, and astrocyte-derived cfDNA in all 29 patients), as opposed to patients without brain metastases or healthy controls. These findings suggest that brain cell type-specific cfDNA methylation markers may enable detection of healthy brain tissue damage resulting from brain metastasis.

#### RNA

Several non-coding RNAs are involved in brain metastases in breast cancer patients. Fu et al. identified circBCBM1 as a proliferation and migration-promoting circRNA in a preclinical breast cancer model and noted that it was markedly upregulated in plasma samples of brain metastatic patients [38]. Another study comparing serum samples from advanced breast cancer patients with and without brain metastasis identified miR-4428 and miR-4480, which detected presence of brain metastasis with an AUC of 0.78 [113]. Additionally, serum miR-330-3p levels were also found to be significantly higher in NSCLC patients with brain metastasis than in those without brain metastasis [129]. These studies indicate that non-coding RNAs may serve as diagnostic biomarkers for brain metastases in breast cancer. No studies have been performed on the value of non-coding RNAs in treatment response of brain metastasis.

Regarding the previously mentioned circulating nucleic acids, thus far only somatic mutation analysis of plasma cfDNA may provide value for diagnosing brain metastases and, potentially, for monitoring treatment response. Brain tissue specific cfDNA methylation patterns in blood appear to be promising, as these are directly linked to brain metastasis and not to systemic metastasis.

## Extracellular vesicles

Extracellular vesicles (EVs) are defined as particles that are actively released from cells, delimited by a lipid bilayer and cannot replicate on their own [132]. They have been found in most biofluids, including blood, saliva, urine, ascites, and CSF, and are released by all cell types, although the majority originates from circulating blood platelets [33, 63]. The EVs are essential for intercellular signaling in (patho)physiological processes, including cancer. EVs are heterogeneous and have distinct biological functions based on their molecular composition, structural characteristics and size, ranging from small EVs of 30 nm to large EVs with a size up to 10 µm [51]. Tumor-derived EVs contribute to different stages of cancer development by promoting and regulating both tumor cells and the tumor microenvironment [78].

EVs are investigated as tumor biomarkers as they bear various useful properties. Due to their envelope, the content of EVs is relatively resistant to degradation. Additionally, they may be able to cross an intact BBB as shown in a mouse model with xenografted human glioma stem cells [41]. As EVs are shed by all cell types, they are believed to reflect the full molecular properties of heterogeneous tumors such as gliomas and their cell of origin [76]. They also contain a plethora of biomolecules, such as DNA, RNA, proteins, and lipids, allowing for multimodal biomarker testing. Here, we cover the brain tumor monitoring potential of EVs.

### EVs in glioma

The release of EVs by gliomas was first reported in 2008 by Skog et al., who found that EVs contain tumor-specific RNAs [118]. Normal cells were shown to sequester these EVs, which could also be isolated from serum. Even without investigating the contents of EVs, their numbers can be informative. A 5.5-fold increase in total plasma EV concentration was observed in 101 glioblastoma patients compared to 29 age-matched healthy controls [105]. Within the glioblastoma group, increased EV plasma levels were significantly associated with shorter survival. EV levels correlated with MR imaging FLAIR hyperintensity volume, but not with T1 contrast-enhancing volume. FLAIR hyperintensity in glioma signifies diffuse infiltration and edema, suggesting increased permeability of the BBB and consequently elevated EV levels. Alternatively, the peritumoral cells may release additional EVs due to tumor-induced brain edema. Importantly, EV concentration did not correlate with platelet counts, indicating that the mechanism causing increased EVs is distinct from disease-associated thrombocytosis.

EV plasma concentrations may also reflect treatment response. In a subset of 34 patients with glioblastoma, EV levels decreased more in patients that underwent gross total resection compared to subtotal tumor resection. During the stable disease phase, EV concentrations remained low, whereas levels rose upon tumor progression. In two of 11 follow-up patients, the EV levels even increased 3–6 months before MR imaging revealed tumor progression, highlighting the potential of this biomarker for glioblastoma monitoring.

Batool et al. have detected several somatic alterations in EV RNA of patients with glioma [15]. They developed a novel ddPCR assay for the detection of EGFRvIII—a glioma specific deletion mutant—in blood plasma, reaching a sensitivity of 73% and a specificity of 98% in a cohort of 40 glioblastoma patients and 14 age-matched healthy controls. The plasma samples were drawn from four patients after glioblastoma treatment, in which EGFRvIII-mutant copies seemed to mirror clinical status. In CSF of three additional patients with recurrent glioblastoma treated with chimeric antigen receptor (CAR) T-cells, the number of mutant copies was concordant with response based on RANO criteria [30]. EGFRvIII was undetectable at time of CAR-T infusion, but increased dramatically in the following weeks, potentially due to treatment-initiated cell death. Subsequently, EGFRvIII became undetectable after treatment. Plasma taken from a single patient before and after infusion showed a decrease to undetectable levels. No plasma or CSF was collected at time of recurrent disease, but given that immunohistochemical analysis of post-treatment tumor tissue was negative for EGFRvIII, increased levels of EGFRvIII-mutant copies in plasma or CSF at tumor recurrence are unlikely. The loss of EGFRvIII through treatment-induced tumor evolution suggests that the detection of a single somatic alteration is not optimal for the monitoring of targeted (immune) therapy. A combination of biomarkers may be preferred.

Analogous to the EGFRvIII assay, the group of Batool et al. developed an assay for the IDH1.R132H mutation [14]. A sensitivity of 75% and specificity of 89% was reached in a cohort of 124 glioma patients (*n* = 80 IDH-mutant) and 9 age-matched healthy controls. Multiple follow-up samples were taken from eight patients with IDH1-mutant glioma. The patients in the disease progression group (*n* = 3; grades 2–4 astrocytoma) and the treatment response group (*n* = 3; subtypes not mentioned) displayed EV RNA-based MAF concordant with disease status. Notably, in the treatment response group, MAF increased at the start of treatment in all three patients, presumably due to increased tumor shedding. In an additional patient, the same initial spike in IDH-mutant MAF was observed, which later returned to baseline. This drop in MAF co-occurred with suspected pseudoprogression, indicating its potential utility in tumor treatment monitoring.

While EV liquid biopsies in their current state are not yet ready for adoption, EVs have promise for monitoring of glioma due to their rich EV cargo, stability and abundance in the blood. The biomarker has undergone significant development, yet a standardized protocol for EV identification is essential before it can be considered ready for large-scale prospective validation studies. As the isolation of EVs is relatively time-consuming, efforts to improve labor intensity are needed to progress its utility as a biomarker.

### EVs in brain metastasis

While studies on EV-associated biomolecules specifically for monitoring brain metastasis are still scarce, growing evidence supports their diagnostic and prognostic utility, with most research focusing on miRNAs and proteins present in EVs.

The screening for brain-metastasis specific miRNAs, Wei et al. conducted RNA-sequencing from plasma-derived EVs and identified 22 differentially expressed miRNAs in plasma of lung cancer patients with and without brain metastasis, of which miR-550a-3-5p was significantly enriched in EVs from patients with brain metastasis [130]. Accordingly, Ruan et al. sequenced RNA of plasma of 42 stage IV breast cancer patients, of which 21 had brain metastasis and found that high levels of miR-199b-5p in EVs are associated with brain metastasis [111].

Additional studies have focused on size and protein cargo of circulating EVs. Carretero-González et al. found that patients with brain metastasis had lower levels of plasma EVs and higher protein concentration in small EVs (sEVs) compared to patients without brain metastasis and healthy controls [28]. Melanoma patients with brain metastasis had decreased STAT3 activation and increased PD-L1 levels in sEVs as compared to patients without brain metastasis, possibly because of systemic immunosuppression in melanoma brain metastasis patients. Rodrigues et al. demonstrated high expression of the cell migration-inducing and hyaluronan-binding protein (CEMIP) in brain metastasis tissue and their secreted EVs in plasma in contrast to tissue from systemic metastasis [110]. Li et al. compared EV-associated proteins in 42 metastatic lung cancer patients, 25 locally advanced lung cancer patients, and 5 healthy controls and identified 120 differentially expressed EV-associated proteins in 28 brain metastatic lung cancer patients, of which MUC5B and SELL could be used as diagnostic biomarkers (AUC 0.774 and 0.720, respectively) [66]. Both miRNA and proteins in plasma EVs are therefore promising biomarkers for brain metastasis.

In addition to aiding in brain metastasis diagnosis, plasma EV content has been linked to disease progression and survival. Chen et al. found that elevated EV-associated integrin β3 levels in 75 lung carcinoma patients who received whole brain radiotherapy for brain metastasis were associated with poorer intracranial control (HR: 1.22 per 1 ng/mL increase; 95% CI 1.012–1.46; *p* = 0.037) and reduced overall survival (HR: 1.15 per 1 ng/mL increase; 95% CI 1.01–1.32; *p* = 0.04) [29]. These results indicate that proteins in EVs, in particular integrin β3, may serve as prognostic biomarkers for brain metastasis.

In conclusion, the diagnostic and prognostic potential of plasma EVs has been demonstrated in brain metastasis patients. Further research is needed to confirm the diagnostic and prognostic role of EVs and to establish whether they can be used for treatment monitoring.

## Platelet RNA

The blood platelets play a significant role in the progression of systemic cancer. Direct and indirect contact between platelets and tumor cells of colon and breast cancer facilitates transition towards a more mesenchymal phenotype, enabling tumors to invade surrounding tissues and metastasize [59]. The platelets even help circulating tumor cells to evade the immune system by forming a physical shield around them [70, 101]. Despite the recognized role of platelets in cancer pathogenesis, platelet counts have inconsistently shown utility as biomarkers in glioma patients [7]. Consequently, research efforts focus on analyzing platelet content rather than platelet counts. Platelets and tumor cells have demonstrated ‘cross-talk’. The transcriptome and proteome of platelets incorporate a tumor signature that includes tumor-specific mutant transcripts [55, 91, 94]. Possibly, this occurs via alternative splicing of pre-mRNAs from megakaryocytes and sequestration of (circulating) tumor-derived RNA molecules. As platelets have a life span of ~ 7 to 10 days, they may provide a real-time snapshot of the tumor status. Additionally, they are practical biomarker candidates: platelets are widely abundant, can be isolated from only 4–6 mL of whole blood, and the isolation procedure is simple. Before processing, whole blood can be stored for as long as 48 h at room temperature, enabling for sample shipment [19].

Over the last decade, our group has developed a platelet mRNA biomarker platform that enables distinction between cancer, non-neoplastic disease, and healthy controls with high accuracy [19–21, 47]. In a cohort of 126 patients with one or multiple brain metastases primarily from NSCLC (*n* = 85), and 89 patients with glioblastoma, we demonstrated that platelet RNA profiles can be used to discriminate brain metastases from glioblastoma with an AUC of 0.84 (95% CI 0.76–0.92; *p* < 0.001) [119]. A subsequent study investigating patients with 18 different tumor types showed that RNA profiles of 93 patients with brain metastasis were distinct to profiles of 299 patients with a similar primary tumor without brain metastasis. Furthermore, platelet RNA profiles of brain metastasis patients had similarities to those of patients with gliomas, suggesting that the platelet transcriptome is influenced by both the primary tumor and the metastatic site [47].

The platelets may also be of value for glioblastoma treatment monitoring. Distinguishing true progression from pseudoprogression, a platelet RNA-based glioblastoma-specific classifier has demonstrated an AUC of 0.86 (95% CI 0.70–1.00; *p* < 0.012) [119]. In a follow-up cohort of 48 glioblastoma patients, the classifier score seemed to mirror disease course, although the correlation varied across patients. In some patients, the score correctly indicated tumor progression before radiological progression occurred. These properties render platelet RNA potentially valuable in therapy monitoring, which is currently being evaluated in the multicenter, prospective PREDICT-study.

A disadvantage of the use of platelet RNA as a biomarker source is that the mechanism of platelet ‘education’ is not completely identified. Recent findings by Karp et al. suggest that platelets are indirectly influenced by other circulating cells rather than sequestering tumor transcripts [53]. Conversely, previous studies have demonstrated transfer of EGFRvIII RNA from glioblastoma to platelets, and RNA transfer between platelets and other cells [42, 61, 94, 107]. Future studies should aim to elucidate these contradictions, through improved wet-lab protocols as well as computational cleaning methods. Platelet RNA remains a promising biomarker in the monitoring of brain tumors, warranting large-scale prospective validation studies.

## Circulating tumor cells

Circulating tumor cells (CTCs) originate from primary or metastatic tumor sites and offer a minimally invasive approach to retrieve information about tumor characteristics [68]. Since most tumors metastasize hematogenously, tumor release of CTCs is a proxy of its metastatic potential. However, CTCs require adaptive mechanisms to survive, resulting in low levels in blood, with only a rare subset capable of initiating brain metastases [58]. Recent technological advances, such as immunomagnetic bead methods, microfluidic technologies, and high-throughput sequencing technology, have significantly improved the detection and characterization of CTCs associated with brain tumors [4, 52, 138].

### CTCs in glioma

Compared to cell-free nucleic acids and EVs, the field of CTCs in glioma has advanced less rapidly due to the technical difficulties in enriching CTCs from glioma. As glioma does not express the epithelial cell adhesion molecule (EpCAM) typical for carcinomas, standard EpCAM-based CTC isolation methods cannot be employed. This has resulted in the development of various protocols that employ different markers and isolation methods [43]. Most studies have focused on improving enrichment of glioma CTCs rather than correlating them to clinical status, a purpose for which current methods may not be sufficiently mature. The reported blood detection rates of CTCs in glioma patients range from 20 to 84%, with the number of cells detected in a tube of blood often in single digits [40, 75, 87, 88, 102, 123, 140, 141]. Several studies have investigated the prognostic and monitoring potential of CTCs in glioma, of which we describe here the most recent and the most important ones.

Using a telomerase reverse transcriptase-based assay, Zhang et al. were able to detect CTCs in plasma of 106 glioma patients with 83% sensitivity [141]. The presence of postoperative, but not preoperative CTCs was associated with poor prognosis, and a significant decrease in the number of CTCs following tumor resection was observed. Unfortunately, CTC levels during the chemotherapeutic treatment phase, and thereafter, are not described. In addition, Sullivan et al. report 39% sensitivity in detecting CTCs in plasma of 33 glioblastoma patients [123]. A significant difference in CTC counts was observed between patients with stable disease and patients with disease progression. Notably, all detected CTCs had the mesenchymal subtype, indicating that a molecular transition of glioblastoma cells may be necessary to enter the circulation. Consequently, the blood may only contain a subset of tumor-derived cells, not fully reflecting the heterogeneity of the primary tumor. A further increase in sensitivity might enable CTCs to play a role in glioma disease monitoring.

Although we did not find any studies that formally investigated CTCs as a biomarker in the monitoring of glioma, some studies report a decrease in CTCs after tumor resection and an increase upon progression. More research is needed to determine their monitoring value.

### CTCs in brain metastasis

In systemic metastasis, CTCs have shown utility in treatment response assessment and post-treatment surveillance [35, 122]. Unfortunately, no studies have specifically evaluated CTCs for monitoring brain metastasis. Most research on CTCs has focused on cellular and molecular adaptations that allow them to cross the BBB and blood‐CSF barrier, colonize the brain microenvironment and form brain- or leptomeningeal metastases [58].

The translational research in human cell lines and mouse models has identified specific molecular profiles that differentiate CTCs associated with brain metastasis from those associated with systemic metastases from the same primary tumor [17, 25, 26, 57, 58, 82, 98, 103, 106, 126, 135, 139]. Building on these findings, several studies have investigated CTCs in blood of patients with brain metastasis. In a mixed cohort of brain metastatic patients with NSCLC, breast cancer, or melanoma, Loreth et al. found that CTCs in blood mostly expressed CD74 and CD44, unlike matched brain tumor tissue [71]. This suggests plasticity of CD44 and CD74 expression on CTCs that survive in blood and penetrate the BBB. Aljohani et al. reported mutations in KEAP1-NRF2-ARE pathway genes in CTCs in blood of lung cancer patients with brain metastasis [8]. In melanoma patients, the RPL/RPS-gene signature in CTCs has been linked with the onset of brain metastasis [27]. These characteristics across various primary solid tumors may provide leads for future development of a CTC-based brain metastasis monitoring platform.

Several studies have investigated overall CTC counts as a prognostic tool for brain metastasis. In NSCLC patients with oligo-metastatic brain disease and patients with concurrent systemic metastases, Hanssen et al. found that presence of ≥ 2 and ≥ 5 CTCs/7.5 mL predicted poorer survival [45]. Naito et al. found no association between pre- and post-treatment CTC counts and brain metastasis presence in small-cell lung cancer (SCLC) patients before, during and after receiving chemo(radio)therapy, although only seven patients had brain metastasis [90]. In contrast, the LANDSCAPE trial showed that early CTC clearance in HER2-positive breast cancer patients with brain metastases predicted intracranial response to HER2-directed therapy combined with chemotherapy and overall survival [100]. However, both responders and non-responders showed a decline in CTC count after one cycle, and only 15% of patients had brain-only metastatic disease, suggesting that reduced CTC levels in blood may reflect overall disease control, rather than intracranial tumor control.

In conclusion, CTC-based liquid biopsies are increasingly implemented in various cancer types. However, the applicability for brain tumor monitoring is insufficiently studied. Important steps have been made in differentiating brain metastasis-associated CTCs from systemic metastasis-associated CTCs. Despite this, further research is required to develop a clinically applicable brain metastasis-specific CTC signature. This may pave the way to CTC-based monitoring of brain metastases.

## Role of myeloid cells/monocytes

Currently, liquid biopsies in brain metastasis patients primarily focus on diagnosis and prognosis. There is a critical need to expand their role in monitoring tumor progression, especially since the incidence of cerebral radiation necrosis is rising, which may be hard to distinguish from tumor progression on MR imaging. A recent study proposed an immunosuppressive marker as a surrogate for differentiation between patients with active and inactive brain metastases [121]. Flow cytometry was used to quantify monocytic myeloid-derived suppressor cells (Mo-MDSC) from peripheral blood in 22 patients with biopsy-proven active brain metastasis or radiation necrosis. They concluded that the HLA-Dr-Vnn2 Index could reliably discriminate recurrent brain metastasis from radiation necrosis. The patients with brain metastasis recurrence showed significantly increased CD14 + HLA-DRneg/low Mo-MDSCs and reduced expression of Vnn2 on circulating CD14 + monocytes compared to those with radiation necrosis. This study has set a foundation for further research finding biomarkers for post-treatment brain metastasis monitoring.

## Cerebrospinal fluid as liquid biopsy source

The BBB restricts the release of biomolecules into the bloodstream, making CSF a potentially superior compartment for studying brain-derived biomarkers. However, CSF collection via lumbar puncture is more invasive than venipuncture and not always possible due to the risk of cerebral herniation. While intraventricular devices such as an Ommaya reservoir enables easier repeat sampling, the surgical placement carries risks such as infection and hemorrhage. Despite these challenges, CSF-derived biomarkers for brain cancer monitoring have been studied; we will highlight the most relevant studies.

### Glioma

In glioma patients, several CSF-based biomarkers are being investigated. One promising candidate is D-2 hydroxyglutarate, a metabolite of mutant IDH, whose CSF concentration differs between patients with IDH-mutant and IDH-wild-type gliomas, as well as pre- and post-resection of IDH-mutant tumors [39, 109]. Ongoing studies will show whether this relatively new CSF biomarker is suitable for treatment monitoring of IDH-mutant gliomas.

Furthermore, several studies have shown the potential of liquid biopsies using cfDNA from CSF. Iser et al. demonstrated molecular-based classification of 75% (*n* = 24) of glioblastoma patients and 53% (*n* = 10) of patients with other types of glioma using targeted next-generation sequencing of cfDNA from CSF [49]. They also included patients with recurrent/progressive glioma (*n* = 14). Klinsing et al. evaluated the diagnostic ability of somatic copy number alterations from CSF cfDNA in brain tumor patients, of which six (26%) glioma patients [56]. They were able to differentiate tumor recurrence from other potential causes of deterioration, e.g., postoperative infection, during surveillance in one patient with a previous history of glioma. Interestingly, a recent case-report indicated the value of CSF-based cfDNA methylation analysis using nanopore sequencing for the diagnosis of a difficult-to-diagnose intracranial lesion [120]. Additionally, Afflerbach et al. demonstrated the potential of nanopore sequencing on cfDNA from CSF for the classification of brain tumors [1]. Most patients in their study were children and adolescents (*n* = 91/129) with pediatric brain tumors. Although a minority of included patients were adults, this study highlights the utility of cfDNA Nanopore sequencing for brain tumor monitoring. This approach shows particular promise in the early detection of tumor recurrence and identification of molecular characteristics, as evidenced by two follow-up patients. Several other studies report on the detection of glioma-derived DNA in CSF, showing that it can be used to track tumor evolution, which lays the foundation for CSF-based tumor monitoring in future [56, 80, 108, 116].

### Brain metastasis

In brain metastasis patients, CSF is deemed a more reliable source than plasma for detecting tumor-derived cfDNA [31, 74, 136]. Wu et al. found that CSF cfDNA had significantly higher concordance with brain tumor tissue than plasma cfDNA (99% vs. 67%) in patients with single brain metastasis, although performance was similar in patients with multiple brain metastases [136]. Additionally, Li et al. observed that cfDNA changes in CSF correlate with intracranial response, while plasma cfDNA seems to reflect extracranial disease response [65].

In summary, CSF collection is more invasive than blood, making it a theoretically suboptimal compartment for frequent liquid biopsies. Nevertheless, CSF is anatomically closer to brain tumors and possibly reflects intracranial disease better than plasma. If CSF-based liquid biopsies, despite all drawbacks, reach excellent performance in clinical practice, the added value of CSF-derived biomarkers could overcome these drawbacks.

## Focused ultrasound-enhanced liquid biopsies

An alternative approach to enhance biomarker detection may be to enrich the repertoire of tumor-derived biomolecules in the blood, for example using focused ultrasound (FUS). Originally designed to temporarily open the BBB to enhance delivery of chemotherapeutic agents, the technique was found to increase the blood concentrations of cfDNA by up to 2.6-fold, neuron-derived EVs by 3.2-fold, and brain-specific protein S100b by 1.4-fold [11, 79, 137]. TERT-mutation cfDNA blood plasma levels rose up to 5.6-fold in a cohort of four patients, indicating that focused ultrasound increased tumor-derived cfDNA [137]. With this proof-of-concept, the LIBERATE-trial, in which low-intensity FUS will be used for cfDNA-based liquid biopsy in glioblastoma, has been initiated. Further research, including this trial, should further elucidate the applicability and efficiency of FUS-enhanced liquid biopsies [2].

## Conclusions and considerations for future research and biobanking

**Fig. 1:**
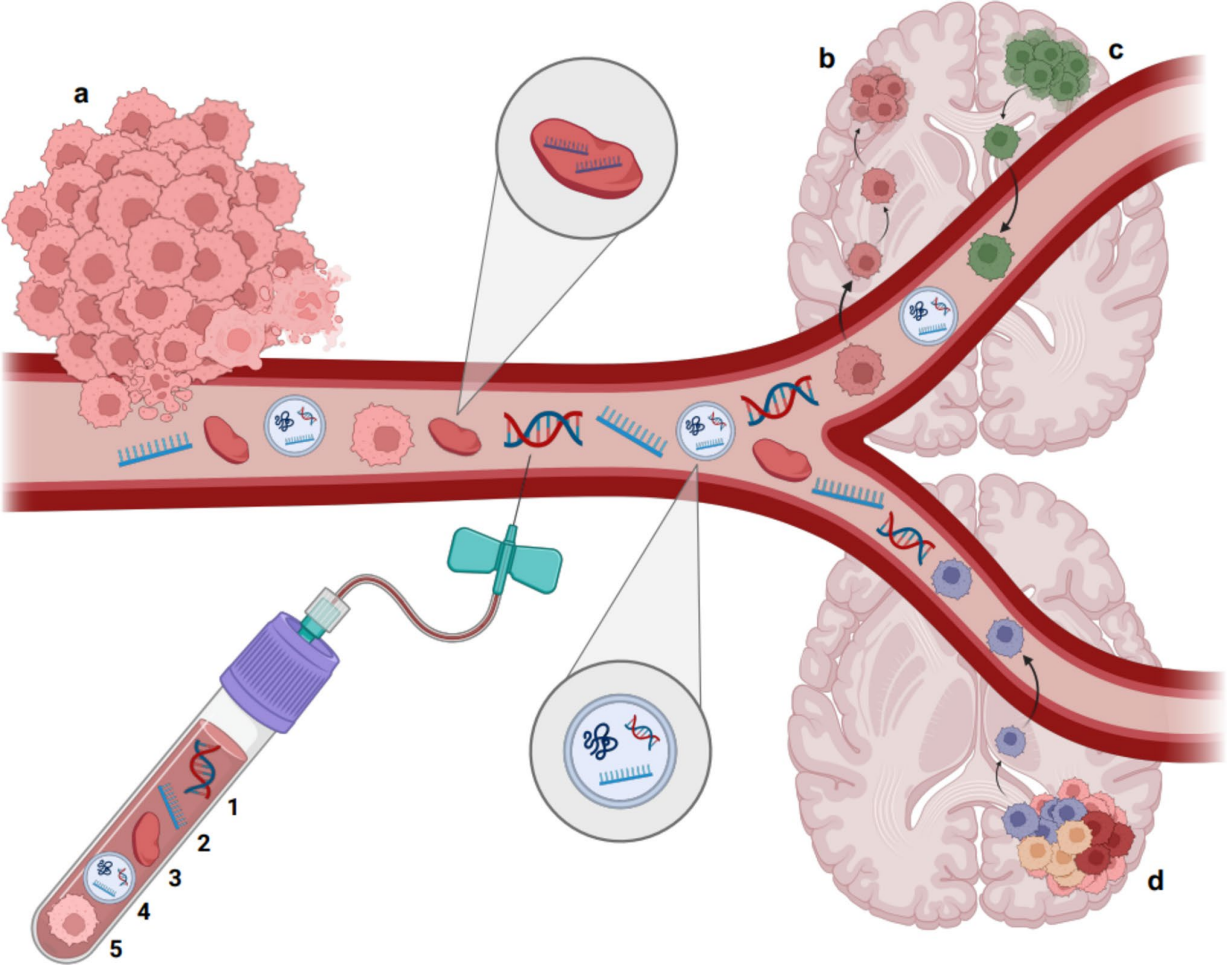
Overview of blood-based biomarkers in glioma and brain metastasis, including ctDNA (1), RNA (2), platelet mRNA (3), extracellular vesicles (4) and circulating tumor cells (5). **a** Primary tumor with cancer cells which intravasate (CTCs), cells secrete EVs, necrotic and apoptotic cells shed nucleic acids (ctDNA, RNA) in circulation; **b** Brain metastasis formation by CTCs; **c** Brain metastasis derived CTCs; **d** Glioma derived CTCs, EVs, ctDNA, RNA

**Fig. 2:**
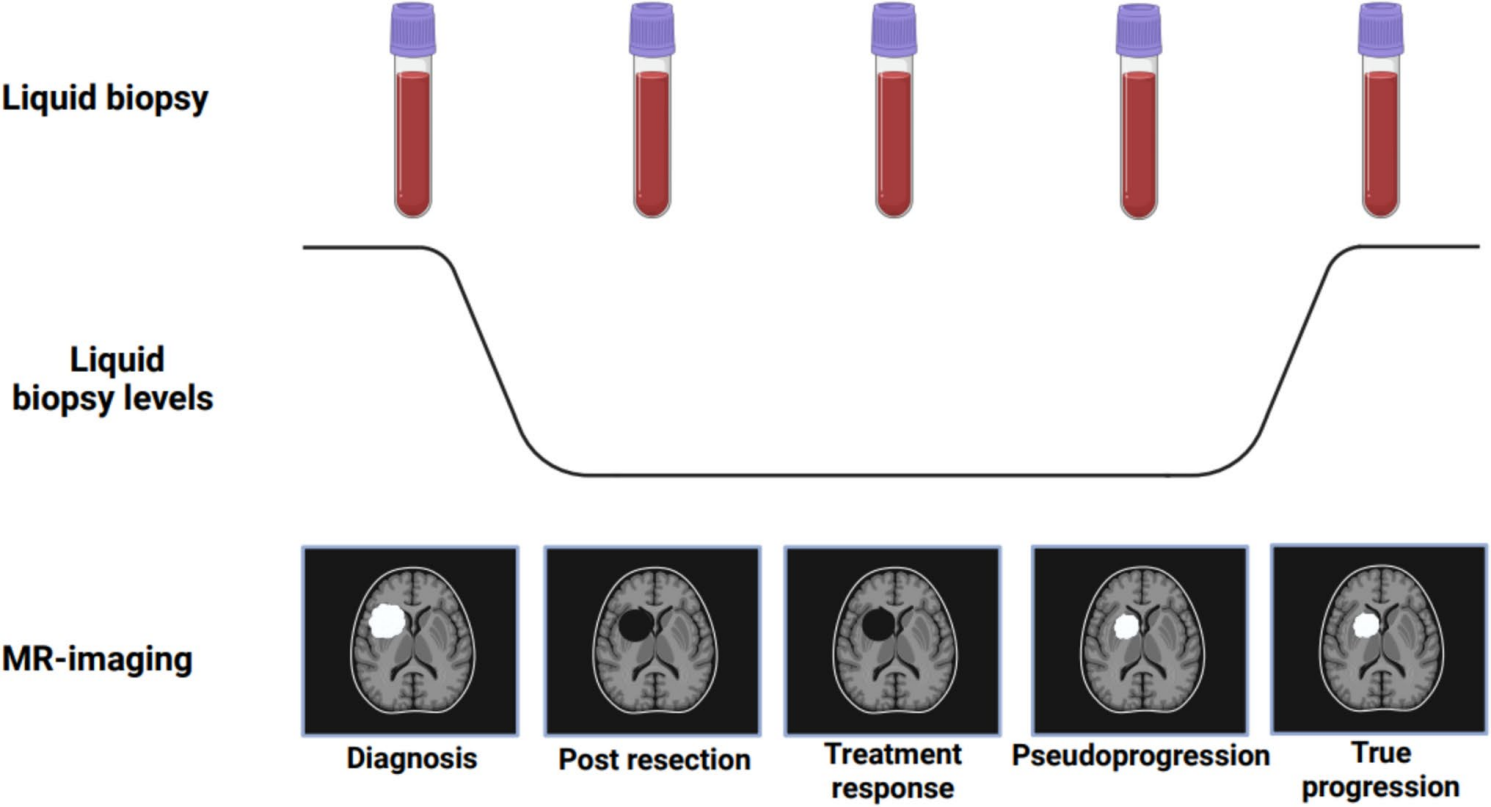
The ideal theoretical liquid biopsy for monitoring treatment response in brain tumors as compared to MR-imaging

The ideal brain tumor biomarker is reliably detectable, stable after collection, and does not require complicated processing (Figs. 1 and 2). Currently, this best describes EVs, miRNA, and platelet RNA (Table 1). Although total cfDNA levels may also prove informative, tumor-derived cfDNA is difficult to detect in blood and repeated CSF collection is less desirable due to its invasive nature. Following cfDNA collection either in blood or CSF tubes, cfDNA has a half-life of less than 1.5 h, making timely processing a challenge. Brain tumor-associated CTC detection in blood is also difficult and time-intensive. In Tables 2 and 3, we provide an overview of the most important studies.

**Table 1:** Overview of analytes in brain tumor liquid biopsies and their monitoring potential

	Advantages	Challenges	Potential for monitoring brain tumors
Circulating nucleic acids (cfDNA, non-coding RNA)	More abundant than CTCs Represents spatial and temporal tumor heterogeneity Contain both genetic and epigenetic information	Short half-life < 1.5h Released by necrotic or apoptotic tumor cells rather than active cells Lower sensitivity in blood vs CSF Only a small fraction is tumor-associated	Non-coding RNAs, especially miRNAs, offer greater stability than cfDNA, and are suggested to have monitoring potential, although further research is required
Extracellular vesicles	Abundant in circulation Lipid envelope protects cargo from degradation Contain various biomolecules, including proteins, DNA, RNA, miRNA Able to cross BBB Represent spatial and temporal tumor heterogeneity Provides insight in tumor intercellular signaling, reflection of tumor microenvironment Detection of metastatic potential	Complex and non-standardized isolation and analysis due to variety of biomolecules Contamination of other cells during isolation Not specific to tumor cells	EVs hold promise for monitoring due to their rich cargo, stability and abundance in the blood, yet efficient and standardized methods for EV isolation and analysis are lacking
Platelet RNA	Highly abundant in circulation Whole blood storage up to 48 h at room temperature Up-to-date information due to limited life span Surrogate profile from multiple local and systemic sources	Mechanism of platelet education is poorly understood Contamination of other cells during isolation may influence platelet transcriptome	Platelet RNA shows promise for monitoring glioma by distinguishing treatment effects from true progression—an essential and challenging aspect of monitoring. The mechanism behind TEPs and their potential in brain metastasis monitoring requires further investigation
Circulating tumor cells	Direct tumor representation, allowing more comprehensive molecular profiling Potential for culture after isolation for further analysis and drug targeting research Developed technique(s) for optimal and standardized isolation	Low abundance of brain tumor CTCs in circulation due to BBB Requires almost immediate processing Significant heterogeneity Limited number of markers for CTC enrichment	CTCs have limited potential for brain tumor monitoring due to low BBB permeability and low abundance in the blood. Although widely studied in other cancers, their applicability in brain tumor monitoring remains insufficiently explored

BBB: blood brain barrier; cfDNA: cell-free DNA; CSF: cerebrospinal fluid; CTCs: circulating tumor cells; EVs: extracellular vesicles; miRNA: microRNA, TEPs: tumor educated platelets

**Table 2:** Clinical studies on liquid biopsies in glioma

Author & year	Population	Source	Biomarker	Findings
*Circulating nucleic acids*				
Bettegowda et al. 2014	Glioma (n=27)	Plasma	cfDNA	Tumor-derived cfDNA was detected in <10% of glioma patients.
Zhi et al. 2015	Astrocytoma (WHO 2007) (n=90) and healthy controls (n=110)	Serum	miRNA	A 9-miRNA panel reached AUC 0.97 in distinguishing astrocytoma from healthy controls. These miRNAs decreased significantly following tumor resection.
Morokoff et al. 2020	Glioma (n=91) and healthy controls (n=17)	Serum	miRNA	A 9-miRNA panel reached AUC 0.998 in the differentiation of glioma and healthy controls. Specific miRNAs correlated with lower-grade glioma and glioblastoma volume on MRI in longitudinal samples of 11 patients. Levels remained stable in two cases of pseudoprogression.
Nassiri et al. 2020	Glioma (n=60)	Plasma	cfDNA	cfDNA methylation profiles: glioma vs other brain tumors and healthy controls: AUC 0.99 IDH-wildtype vs other: AUC 0.71 IDH-mutant vs other: AUC 0.82.
Moulière et al. 2021	Glioblastoma with samples of CSF (n=8), plasma (n=12), and urine (n=8)	CSF, plasma, and urine	cfDNA	Selection of fragmented cfDNA detection sensitivity: CSF 88%; plasma 83%; urine 75%.
Muralidharan et al. 2021	TERT-promoter mutant glioma (n=88)	Plasma	cfDNA	TERT-promoter mutation was detected in 63% of TERT-promoter mutant patients. MRI contrast enhancement volume correlated with MAF.
Sabedot et al. 2021	Glioma (n=149) and other brain tumors (n=86)	Serum	cfDNA	cfDNA methylation profiles distinguished glioma from other brain tumors with 98% accuracy. Progression vs pseudoprogression distinction in 3 patients with glioma.
Afflerbach et al. 2024	Ependymoma (n=17), pilocytic astrocytoma (n=11), and other brain tumors (n=101)	CSF	cfDNA	In 2 follow-up patients, early detection of tumor recurrence and identification of molecular characteristics was possible.
Iser et al. 2024	Glioma (n=51), nonmalignant (n=17), and nondiagnostic (n=17)	CSF	cfDNA	Targeted sequencing of cfDNA enabled classification of 75% of glioblastomas and 53% of other glioma types. 14 recurrent/progressive gliomas were included.
Klinsing et al. 2024	Glioma (n=6)	CSF	cfDNA	In one glioma patient, tumor recurrence was successfully differentiated from other causes of clinical deterioration using somatic copy number alterations.
Iorgulescu et al. 2025	Glioblastoma (n=8)	Plasma	cfDNA	Tumor cfDNA detection with 88% sensitivity. MAESTRO-Pool assay may help distinguish true progression from pseudoprogression in 7 patients.
*Extracellular vesicles*				
Batool et al. 2022	Glioblastoma (n=40) and healthy controls (n=14)	Plasma	EGFRvIII mRNA in EVs	EV EGFRvIII qPCR assay: sensitivity 73%; specificity 98%. Four samples drawn after treatment reflected clinical status.
Batool et al. 2024	Glioma (n=124) of which IDH-mutant (n=80)	Plasma	IDH1.R132H mRNA in EVs	Detection of IDH mutation: sensitivity 75%; specificity 89%. MAF was concordant with disease status in 8 patients.
Choi et al. 2024	Glioblastoma (n=4)	CSF	EGFRvIII mRNA in EVs	EGFRvIII mutant copies reflected clinical status in patients treated with CAR T-cells.
Ricklefs et al. 2024	Glioblastoma (n=101) and healthy controls (n=29)	Plasma	Total EVs	EV levels were 5.5-fold higher in glioblastoma than healthy controls. EV levels correlated with MRI FLAIR volume. EV levels reflected disease status in 11 patients during follow-up.
*Platelet RNA*				
Sol et al. 2020	Glioblastoma (n=89) and BM (n=126)	Plasma	Platelet mRNA panel	mRNA-based classifier in glioblastoma vs. BM: AUC 0.84. True progression vs pseudoprogression: AUC 0.86.
In ‘t Veld et al. 2022	BM (n=93) and similar non-brain tumor (n=299)	Plasma	Platelet mRNA panel	Platelet mRNA of BM patients was distinct to that of non-brain tumors of similar origin and had similarities to that of gliomas.
*Circulating tumor cells*				
Sullivan et al. 2015	Glioblastoma (n=33)	Blood	CTCs	CTCs detected in 39% of patients. Difference in CTCs between stable disease vs. progression.
Zhang et al. 2021	Glioma (n=106)	Blood	CTCs	CTC detection sensitivity: 83%. Decrease in CTCs after resection. Presence of postoperative CTCs associated with poor prognosis.
Qi et al. 2024	Glioma (n=63)	Blood	CTCs and CTMs	Detection of CTCs in 84% of patients. CTCs and CTMs may aid in survival prognostication.
*Other*				
Riviere-Cazaux et al. 2023	IDH-mutant glioma (n=7)	CSF	D-2-hydroxyglutarate	Concentrations of D-2-hydroxyglutarate decreased after resection and were concordant with disease status.

AUC: area under the receiver operating characteristic curve; BM: brain metastasis; cfDNA: cell-free DNA; CSF: cerebrospinal fluid; CTCs: circulating tumor cells; CTMs: circulating tumor microemboli; EV: extracellular vesicle; FLAIR: fluid attenuated inversion recovery; MAF: mutant allele frequency; miRNA: microRNA; MRI: magnetic resonance imaging; mRNA: messenger RNA

**Table 3:** Clinical studies on liquid biopsies in brain metastases

Author & year	Population	Source	Biomarker	Finding
*Circulating nucleic acids*				
Barciszweska et al. 2018	BM (*n* = 45)	Blood	m^5^C	M^5^C content in matched BM tissue and peripheral blood samples were similar and moderately strong correlated
Sato et al. 2019	Breast cancer with BM (*n* = 51)	Serum	miR-4428 and miR-4480	Serum miR-4428 and miR-4480 can distinguish patients with and without BM (AUC both miRNAs: 0.78)
Seremet et al. 2019	Melanoma (*n* = 85), subset with BM (*n* = 31)	Plasma	BRAF^V600^ and NRAS^Q61/G12/G13^ mutations in cfDNA	1. Undetectable tumor-derived cfDNA at baseline is associated with better PFS (HR: 0.47, *p* = 0.01) and OS (HR: 0.37, *p* = 0.005) 2. Undetectable tumor-derived cfDNA during treatment is associated with lower HR of death (adjusted-HR: 0.16, *p* < 0.001)
Wei et al. 2019	NSCLC (*n* = 122), subset with BM (*n* = 62)	Serum	miR-330-3p	Serum miR-330-3p levels are significantly higher in NSCLC patients with BM than in those without BM
Aldea et al. 2020	Advanced NSCLC (*n* = 247)	Plasma	cfDNA	CfDNA was detected in 52% of patients with isolated CNS disease vs 84% in no CNS disease and 92% in concurrent CNS and systemic disease, with lower detection of driver mutations (37% vs 77% and 73%, respectively) and resistance mutations (6% versus 45% and 44%)
Lee et al. 2020	Melanoma with BM (*n* = 72) subset with oligo-BM (*n* = 13)	Plasma	cfDNA	Undetectable tumor-derived cfDNA is associated with extracranial response and improved survival at baseline (HR: 0.51, *p* = 0.03) and during treatment (HR: 0.32, *p* ≤ 0.01)
Liang et al. 2020	Glioma (*n* = 21) and BM (*n* = 7)	Blood	cfDNA	CfDNA was detected in blood in 2/7 BM patients (28.3%). Genetic alterations in ALK, MDM2, ATM, BRCA1, FGFR1 and KRAS were associated with BM
Fu et al. 2021	Breast cancer (*n* = 40), subset with BM (*n* = 20)	Plasma	circRNA	Significant upregulation of circBCBM1 in plasma samples of BM patients with BM compared to patients without BM
Kim et al. 2022	NSCLC stage IV (*n* = 311)	Plasma	cfDNA	Significant association between positive plasma EGFR mutation status and BM (adjusted-OR, 2.73; *p* = 0.003)
Pangeni et al. 2022	Breast cancer (*n* = 30)	Serum	miR124-2, CCDC8 and miR3193	Hypermethylation of miR124-2 and CCDC8 and hypomethylation of miR3193 is associated with BM
Alder et al. 2024	BM of solid tumors (*n* = 253)	Serum	cfDNA	1. Genomic alterations MAFs ≥ 1% and median MAF percentage per patient were higher in patients with concurrent intra- and extracranial disease (80.6%, median 4.75) and isolated extracranial progressive disease (73.4%, median 2.55) compared to isolated intracranial progressive disease (65.5%, median 1.6) 2. In breast cancer, ESR1 and PIK3CA mutations were most frequent, while in lung cancer, EGFR mutations were most common in the isolated intracranial progression group compared to isolated extracranial or concurrent intra- and extracranial progression."
Zuccato et al. 2024	Lung cancer (*n* = 346)	Plasma	cfDNA methylation	Non-invasive methylome-based cfMeDIP–seq can identify and confirm BM with high accuracy (AUC: 0.80, 95%-CI 0.68–0.93)
*Extracellular vesicles*				
Rodrigues et al. 2019	Breast and lung cancer (*n* = 278)	Plasma	Exosomal CEMIP protein	Elevated exosomal protein CEMIP is associated with BM
Chen et al. 2021	Lung cancer with BM (*n* = 75)	Plasma	Exosomal integrin β3	Elevated exosomal Integrin β3 is associated with intracranial disease progression and worse overall survival following whole brain radiotherapy
Wei et al. 2021	Lung cancer with (*n* = 3) and without (*n* = 3) BM	Plasma	Exosomal miR-550a-3-5p	Significant upregulation of miR-550a-3-5p in the EVs of BM patients compared to patients without BM
Carretero-González et al. 2022	BM (*n* = 42), cancer without BM (*n* = 50), and healthy controls (*n* = 32)	Plasma	Exosomal protein cargo; STAT3; PD-L1	1. Lower number of circulating EVs and higher protein concentration in small EVs in BM patients is associated with aggressive clinical course and worse survival 2. Increased STAT3 activation in BM breast cancer patients and decreased STAT3 and increased PD-L1 activation in BM melanoma patients
Li et al. 2023	Lung cancer with BM (*n* = 26), with locally advanced disease (*n* = 25), and healthy controls (*n* = 5)	Plasma	MUC5B; SELL	Exosomal MUC5B and SELL can differentiate BM from healthy controls and locally advanced disease (AUC: 0.774 and 0.720, respectively)
Ruan et al. 2024	Stage IV breast cancer (*n* = 42) with (*n* = 21) and without (*n* = 21) BM	Serum	Exosomal miR-199b-5p	Exosomal miR-199b-5p is significantly higher in stage IV breast cancer patients with BM compared to patients without BM
*Platelet RNA*				
Sol et al. 2020	Glioblastoma (*n* = 89) and BM (*n* = 126)	Plasma	Platelet mRNA	Platelet mRNA profiles achieve high diagnostic accuracy in differentiating glioblastoma from BM (AUC: 0.96, 0.89, and 0.84 across training, evaluation, and validation series, respectively)
In ‘t Veld et al. 2022	Glioma (*n* = 132), BM (*n* = 93), and systemic metastatic cancer (*n* = 299)	Plasma	Platelet mRNA	Platelet mRNA profiles enable diagnostic differentiation of primary brain cancers from extracranial cancers, with BM mRNA levels clustering in between mRNA levels of primary brain cancers and extracranial cancers
*Circulating tumor cells*				
Naito et al. 2012	SCLC (*n* = 51)	Blood	CTC count	No association between BM and blood CTC levels at baseline, post-treatment and intracranial tumor relapse
Pierga et al. 2013	HER2-positive metastatic breast cancer (*n* = 44)	Blood	CTC count	Early clearance of CTCs is associated with BM response to targeted therapy
Aljohani et al. 2018	Metastatic melanoma, breast, and colon cancer (*n* = 10)	Blood	CTCs; mutations in Keap1-Nrf2-ARE pathway	Mutations in KEAP1-NRF2-ARE pathway genes in CTCs may provide survival advantage to CTCs allowing their dissemination to distant organs, including BM
Hanssen et al. 2018	NSCLC with BM (*n* = 52)	Blood	CTC count	Both ≥ 2 CTCs/ 7.5 mL and ≥ 5 CTCs/7.5 mL blood are independent prognostic indicators for shorter overall survival time in patients with BM (≥ 2CTCs/7.5 mL ≥ HR: 4.694, *p* = 0.004, CI 1.650–13.354, and ≥ 5 CTCs/7.5 mL ≥ HR: 4.963, *p* = 0.003, CI 1.752–14.061)
Loreth et al. 2021	Breast (*n* = 14), NSCLC (*n* = 18), melanoma (*n* = 11) with BM	Blood	CTCs; CD44, CD74	CD74 and CD44 expression on CTCs is associated with BM
Bowley et al. 2022	Melanoma (*n* = 7)	Blood	CTCs; RPL/RPS	Overexpression of RPL/RPS gene signature in melanoma CTCs is associated with onset of BM
*Other*				
Soler et al. 2021	Stage IV cancer with BM (*n* = 22)	Blood	Mo-MDSC	The DVI reliably discriminates recurrent BM from radiation necrosis based upon the combined expression levels of HLA-DR and VNN2 among CD14^+^ Mo-MDSC
Lubotzky et al. 2022	Cancer without BM (*n* = 113), with BM (*n* = 29), and healthy controls (*n* = 127)	Plasma	Brain-derived cfDNA	cfDNA levels from all three brain cell types (neurons, astrocytes, oligodendrocytes) are significantly higher in cancer patients with BM compared with cancer patients (localized tumor and metastatic combined) without BM or healthy controls

ALK: anaplastic lymphoma kinase; ARE: antioxidant response elements; ATM: ataxia telangiectasia mutated; AUC: area under the receiver operating characteristic curve; BM: brain metastases; BRCA1: breast cancer gene 1; CEMIP: cell migration inducing hyaluronidase 1; cfDNA: cell-free DNA; cfMeDIP-seq: cell-free methylated DNA immunoprecipitation and high-throughput sequencing; CI confidence interval; circRNA: circular RNA; CNS: central nervous system; CTCs: circulating tumor cells; DVI: DR-Vanin Index; EVs: extracellular vesicles; FGFR1: fibroblast growth factor receptor 1; HER-2: human epidermal growth factor receptor 2; HR: hazard ratio; KEAP1: kelch-like ECH-associated protein 1; KRAS: Kirsten rat sarcoma virus; MAF: mutant allele frequency; MDM2: murine double minute-2; miRNA: microRNA; Mo-MDSC: Monocytic Myeloid-Derived Suppressor Cells; mRNA: messenger RNA; m^5^C: 5-methylcytosine; MUC5B: mucin-5B; NRF2: nuclear factor erythroid-2 related factor 2; NSCLC: non-small cell lung cancer; OR: odds ratio; PD-L1: programmed cell death ligand 1; RPL: ribosomal proteins large; RPS: ribosomal protein small; SCLC: small cell lung cancer; STAT3: signal transducer and activator of transcription 3; SELL: selectin L; VNN2: vascular non-inflammatory molecule 2

The molecular information obtained from liquid biopsies could complement current clinical, histopathological, tissue molecular and imaging diagnostics tests. Despite extensive research on liquid biopsy in brain tumors over the past decades, no platform is ready for clinical implementation. Progression monitoring using liquid biopsy in neuro-oncological diseases is still in its infancy, with most studies pursuing diagnostic or prognostic goals only. Studies assessing diffuse gliomas show primarily potential in the higher-grade tumors, whereas lung and breast cancer are the most frequent tumor types assessed in the context of brain metastases. The sampling time points are often limited to pre- and post-resection in glioma and to the first weeks of systemic treatment in brain metastasis. We foresee the main clinical applicability of liquid biopsies in brain tumors to be therapy monitoring, with markers reflecting treatment response, especially when treatment-related effects (pseudoprogression/radionecrosis) are suspected. Treatment-naïve detection of brain tumor-associated biomarkers is a prerequisite for future therapy-monitoring and aligns with numerous studies searching for diagnostic markers. However, the next step—evaluating monitoring potential of liquid biopsies—is a significant challenge. It requires longitudinally collected samples, extensive patient and sample annotation, and sufficient research funds. To demonstrate clinical potential, biomarkers subsequently require validation in large, prospective, preferably multicenter cohorts. The final step towards clinical implementation is an interventional trial to confirm that the proposed biomarker informs clinical decisions including treatment continuation in case of pseudoprogression/radionecrosis or alternative treatment options in case of true tumor progression. MR imaging, with all its modalities, may be complemented by liquid biopsies.

A prospective biomarker validation study should be meticulously planned to ensure proper evaluation of performance in a clinical context. For a fair comparison, time points of the investigational test should be harmonized as much as possible with the gold standard test. For a progression versus pseudoprogression design, this would mean collecting blood at the moment of MR imaging evaluation. Moreover, a well-defined ‘gold standard’ outcome measure should be selected, such as RANO-evaluation of imaging, clinical characteristics, and/or systematically collected tissue biopsies [69, 133]. The clinical feasibility of the investigational test needs to be considered, and its clinical relevance should be defined. When studying tumor molecular evolution, sample collection is preferred when this will have consequences for treatment, including potential inclusion in experimental studies. The results of a biomarker test should be included in the multidisciplinary decision about the patient’s treatment, jointly made by neurologists, neurosurgeons, medical oncologists, radiotherapists, and pathologists.

When collecting samples, the costs for patients and institutions need to be weighed against the potential benefits of isolating multiple markers at various time points. Incorporating a broader range of markers allows for multimodal platforms that combine a panel of biomarkers with the purpose of increasing sensitivity and specificity. As some analytes require rapid processing after collection, the choice of biomarkers should also depend on expected logistics. The measurement of unstable biomolecules can be unfeasible in multicenter research, especially if the samples need to be transported to a central processing facility. The preservatives exist for a subset of biomolecules but amount to an extra processing step and additional costs. Furthermore, following the wide adoption of techniques such as next-generation sequencing, machine learning can play a significant role in interpreting extensive datasets and drawing meaningful conclusions.

Finally, simple, standardized protocols should be used as much as possible for each analyte for the benefit of reusability, comparability and clinical adoption. Ideally, the protocol can be performed in a standard diagnostic laboratory in non-academic hospitals of a medium size. To preserve valuable samples, leftover material should be kept in a biobank for long-term storage and reuse in future studies. We encourage world- or nationwide initiatives in central registration of samples to make maximal use of collected samples and avoid unnecessary spending of resources collecting new samples. An example of such combined efforts is the Brain-Liquid Biopsy Consortium [16, 117].

In conclusion, liquid biopsies have the potential to monitor progression of brain cancer using novel molecular biomarkers, and to inform treatment decisions. To take full advantage of this potential, focus will need to shift to large-scale prospective validation, preferably in a collaborative manner. Well-considered choices need to be made when designing these studies to ensure maximum impact.

## Methods

A comprehensive PubMed search was conducted from its inception in 1996 through January 15, 2025. Language was restricted to English. Key search terms included synonyms of “liquid biopsy” and biomarker subtypes, such as “cfDNA”, extracellular vesicles”, and “circulating tumor cells”, as well as “pseudo-progression”, "radionecrosis", “glioma” and “brain metastases”. The references from identified studies were reviewed for any additional relevant research.

## References

[1] Afflerbach AK, Rohrandt C, Brändl B, Sönksen M, Hench J, Frank S et al (2024) Classification of brain tumors by nanopore sequencing of cell-free DNA from cerebrospinal fluid. Clin Chem 70:250–260. 10.1093/clinchem/hvad115 37624932 10.1093/clinchem/hvad115

[2] Ahluwalia MS, Ozair A, Sahgal A, Burns TC, Groot JFd, Mogilner A et al (2024) A prospective, multicenter trial of low-intensity focused ultrasound (LIFU) for blood-brain barrier disruption for liquid biopsy in glioblastoma (LIBERATE). J Clin Oncol. 10.1200/JCO.2024.42.16_suppl.TPS209838608213

[3] Aizer AA, Lamba N, Ahluwalia MS, Aldape K, Boire A, Brastianos PK et al (2022) Brain metastases: a society for neuro-oncology (SNO) consensus review on current management and future directions. Neuro Oncol 24:1613–1646. 10.1093/neuonc/noac118 35762249 10.1093/neuonc/noac118 PMC9527527

[4] Akpe V, Kim TH, Brown CL, Cock IE (2020) Circulating tumour cells: a broad perspective. J R Soc Interface 17:20200065. 10.1098/rsif.2020.0065

[5] Aldea M, Hendriks L, Mezquita L, Jovelet C, Planchard D, Auclin E et al (2020) Circulating tumor DNA analysis for patients with oncogene-addicted NSCLC With isolated central nervous system progression. J Thorac Oncol 15:383–391. 10.1016/j.jtho.2019.11.024 31843682 10.1016/j.jtho.2019.11.024

[6] Alder L, Broadwater G, Green M, Van Swearingen AED, Lipp ES, Clarke JM et al (2024) Unique genomic alterations in the circulating tumor DNA of patients with solid tumors brain metastases. Neurooncol Adv. 10.1093/noajnl/vdae052 38680992 10.1093/noajnl/vdae052 PMC11046982

[7] Ali H, Harting R, de Vries R, Ali M, Wurdinger T, Best MG (2021) Blood-based biomarkers for glioma in the context of gliomagenesis: a systematic review. Front Oncol 11:665235. 10.3389/fonc.2021.665235 34150629 10.3389/fonc.2021.665235 PMC8211985

[8] Aljohani HM, Aittaleb M, Furgason JM, Amaya P, Deeb A, Chalmers JJ et al (2018) Genetic mutations associated with lung cancer metastasis to the brain. Mutagenesis 33:137–145. 10.1093/mutage/gey003 29474635 10.1093/mutage/gey003 PMC6454536

[9] Arroyo JD, Chevillet JR, Kroh EM, Ruf IK, Pritchard CC, Gibson DF et al (2011) Argonaute2 complexes carry a population of circulating microRNAs independent of vesicles in human plasma. Proc Natl Acad Sci 108:5003–5008. 10.1073/pnas.1019055108 21383194 10.1073/pnas.1019055108 PMC3064324

[10] Bagley SJ, Nabavizadeh SA, Mays JJ, Till JE, Ware JB, Levy S et al (2020) Clinical utility of plasma cell-free DNA in adult patients with newly diagnosed glioblastoma: a pilot prospective study. Clin Cancer Res 26:397–407. 10.1158/1078-0432.Ccr-19-2533 31666247 10.1158/1078-0432.CCR-19-2533 PMC6980766

[11] Bakker A, Ixkes AE, Venugopal H, Ries MG, Lak NSM, de Vos F et al (2024) Focused ultrasound-enhanced liquid biopsy: a promising diagnostic tool for brain tumor patients. Cancers (Basel). 10.3390/cancers16081576 38672658 10.3390/cancers16081576 PMC11049441

[12] Barciszewska AM (2018) Global DNA demethylation as an epigenetic marker of human brain metastases. Biosci Rep. 10.1042/bsr20180731 10.1042/BSR20180731 PMC6200709 30254100

[13] Barnholtz-Sloan JS, Sloan AE, Davis FG, Vigneau FD, Lai P, Sawaya RE (2004) Incidence proportions of brain metastases in patients diagnosed (1973 to 2001) in the Metropolitan Detroit Cancer Surveillance System. J Clin Oncol 22:2865–2872. 10.1200/jco.2004.12.149 15254054 10.1200/JCO.2004.12.149

[14] Batool SM, Escobedo AK, Hsia T, Ekanayake E, Khanna SK, Gamblin AS et al (2024) Clinical utility of a blood based assay for the detection of IDH1.R132H-mutant gliomas. Nature Commun 15:7074. 10.1038/s41467-024-51332-7 39152110 10.1038/s41467-024-51332-7 PMC11329733

[15] Batool SM, Muralidharan K, Hsia T, Falotico S, Gamblin AS, Rosenfeld YB et al (2022) Highly sensitive EGFRvIII detection in circulating extracellular vesicle RNA of glioma patients. Clin Cancer Res 28:4070–4082. 10.1158/1078-0432.Ccr-22-0444 35849415 10.1158/1078-0432.CCR-22-0444 PMC9475243

[16] Batool SM, Yekula A, Khanna P, Hsia T, Gamblin AS, Ekanayake E et al (2023) The liquid biopsy consortium: challenges and opportunities for early cancer detection and monitoring. Cell Rep Med 4:101198. 10.1016/j.xcrm.2023.101198 37716353 10.1016/j.xcrm.2023.101198 PMC10591039

[17] Berghoff AS, Liao Y, Karreman MA, Ilhan-Mutlu A, Gunkel K, Sprick MR et al (2021) Identification and characterization of cancer cells that initiate metastases to the brain and other organs. Mol Cancer Res 19:688–701. 10.1158/1541-7786.Mcr-20-0863 33443114 10.1158/1541-7786.MCR-20-0863 PMC9281611

[18] Berghoff AS, Schur S, Füreder LM, Gatterbauer B, Dieckmann K, Widhalm G et al (2016) Descriptive statistical analysis of a real life cohort of 2419 patients with brain metastases of solid cancers. ESMO Open 1:e000024. 10.1136/esmoopen-2015-000024 27843591 10.1136/esmoopen-2015-000024 PMC5070252

[19] Best MG, In ‘t Veld SGJG, Sol N, Wurdinger T (2019) RNA sequencing and swarm intelligence–enhanced classification algorithm development for blood-based disease diagnostics using spliced blood platelet RNA. Nat Protoc 14:1206–1234. 10.1038/s41596-019-0139-5 30894694 10.1038/s41596-019-0139-5

[20] Best MG, Sol N, Int Veld S, Vancura A, Muller M, Niemeijer AN et al (2017) Swarm intelligence-enhanced detection of non-small-cell lung cancer using tumor-educated platelets. Cancer Cell 32:238-252.e239. 10.1016/j.ccell.2017.07.004 28810146 10.1016/j.ccell.2017.07.004 PMC6381325

[21] Best Myron G, Sol N, Kooi I, Tannous J, Westerman Bart A, Rustenburg F et al (2015) RNA-seq of tumor-educated platelets enables blood-based pan-cancer, multiclass, and molecular pathway cancer diagnostics. Cancer Cell 28:666–676. 10.1016/j.ccell.2015.09.018 26525104 10.1016/j.ccell.2015.09.018 PMC4644263

[22] Best MG, Sol N, Zijl S, Reijneveld JC, Wesseling P, Wurdinger T (2015) Liquid biopsies in patients with diffuse glioma. Acta Neuropathol 129:849–865. 10.1007/s00401-015-1399-y 25720744 10.1007/s00401-015-1399-y PMC4436687

[23] Bettegowda C, Sausen M, Leary RJ, Kinde I, Wang Y, Agrawal N et al (2014) Detection of circulating tumor DNA in early- and late-stage human malignancies. Sci Transl Med. 10.1126/scitranslmed.3007094 24553385 10.1126/scitranslmed.3007094 PMC4017867

[24] Boisselier B, Gállego Pérez-Larraya J, Rossetto M, Labussière M, Ciccarino P, Marie Y et al (2012) Detection of IDH1 mutation in the plasma of patients with glioma. Neurology 79:1693–1698. 10.1212/WNL.0b013e31826e9b0a 23035067 10.1212/WNL.0b013e31826e9b0a

[25] Boral D, Vishnoi M, Liu HN, Yin W, Sprouse ML, Scamardo A et al (2017) Molecular characterization of breast cancer CTCs associated with brain metastasis. Nat Commun 8:196. 10.1038/s41467-017-00196-1 28775303 10.1038/s41467-017-00196-1 PMC5543046

[26] Bos PD, Zhang XH, Nadal C, Shu W, Gomis RR, Nguyen DX et al (2009) Genes that mediate breast cancer metastasis to the brain. Nature 459:1005–1009. 10.1038/nature08021 19421193 10.1038/nature08021 PMC2698953

[27] Bowley TY, Lagutina IV, Francis C, Sivakumar S, Selwyn RG, Taylor E et al (2022) The RPL/RPS gene signature of melanoma CTCs associates with brain metastasis. Cancer Res Commun 2:1436–1448. 10.1158/2767-9764.crc-22-0337 36407834 10.1158/2767-9764.CRC-22-0337 PMC9668078

[28] Carretero-González A, Hergueta-Redondo M, Sánchez-Redondo S, Ximénez-Embún P, Manso Sánchez L, Gil EC et al (2022) Characterization of plasma circulating small extracellular vesicles in patients with metastatic solid tumors and newly diagnosed brain metastasis. Oncoimmunology 11:2067944. 10.1080/2162402x.2022.2067944 35481283 10.1080/2162402X.2022.2067944 PMC9037466

[29] Chen GY, Cheng JC, Chen YF, Yang JC, Hsu FM (2021) Circulating exosomal integrin β3 is associated with intracranial failure and survival in lung cancer patients receiving cranial irradiation for brain metastases: a prospective observational study. Cancers (Basel). 10.3390/cancers13030380 33498505 10.3390/cancers13030380 PMC7864205

[30] Choi BD, Gerstner ER, Frigault MJ, Leick MB, Mount CW, Balaj L et al (2024) Intraventricular CARv3-TEAM-E T cells in recurrent glioblastoma. N Engl J Med 390:1290–1298. 10.1056/NEJMoa2314390 38477966 10.1056/NEJMoa2314390 PMC11162836

[31] De Mattos-Arruda L, Mayor R, Ng CKY, Weigelt B, Martínez-Ricarte F, Torrejon D et al (2015) Cerebrospinal fluid-derived circulating tumour DNA better represents the genomic alterations of brain tumours than plasma. Nat Commun 6:8839. 10.1038/ncomms9839 26554728 10.1038/ncomms9839 PMC5426516

[32] Diehl F, Schmidt K, Choti MA, Romans K, Goodman S, Li M et al (2008) Circulating mutant DNA to assess tumor dynamics. Nat Med 14:985–990. 10.1038/nm.1789 18670422 10.1038/nm.1789 PMC2820391

[33] Doyle LM, Wang MZ (2019) Overview of extracellular vesicles, their origin, composition, purpose, and methods for exosome isolation and analysis. Cells 8:727 31311206 10.3390/cells8070727 PMC6678302

[34] Ellingson BM, Chung C, Pope WB, Boxerman JL, Kaufmann TJ (2017) Pseudoprogression, radionecrosis, inflammation or true tumor progression? challenges associated with glioblastoma response assessment in an evolving therapeutic landscape. J Neurooncol 134:495–504. 10.1007/s11060-017-2375-2 28382534 10.1007/s11060-017-2375-2 PMC7893814

[35] Fehm T, Mueller V, Banys-Paluchowski M, Fasching PA, Friedl TWP, Hartkopf A et al (2024) Efficacy of Lapatinib in Patients with HER2-Negative Metastatic Breast Cancer and HER2-Positive Circulating Tumor Cells-The DETECT III Clinical Trial. Clin Chem 70:307–318. 10.1093/clinchem/hvad144 38175595 10.1093/clinchem/hvad144

[36] Fleischhacker M, Schmidt B (2007) Circulating nucleic acids (CNAs) and cancer–a survey. Biochim Biophys Acta 1775:181–232. 10.1016/j.bbcan.2006.10.001 17137717 10.1016/j.bbcan.2006.10.001

[37] Fontanilles M, Marguet F, Beaussire L, Magne N, Pépin LF, Alexandru C et al (2020) Cell-free DNA and circulating TERT promoter mutation for disease monitoring in newly-diagnosed glioblastoma. Acta Neuropathol Commun 8:179. 10.1186/s40478-020-01057-7 33148330 10.1186/s40478-020-01057-7 PMC7641818

[38] Fu B, Liu W, Zhu C, Li P, Wang L, Pan L et al (2021) Circular RNA circBCBM1 promotes breast cancer brain metastasis by modulating miR-125a/BRD4 axis. Int J Biol Sci 17:3104–3117. 10.7150/ijbs.58916 34421353 10.7150/ijbs.58916 PMC8375234

[39] Fujita Y, Nunez-Rubiano L, Dono A, Bellman A, Shah M, Rodriguez JC et al (2022) IDH1 *p*. R132H ctDNA and D-2-hydroxyglutarate as CSF biomarkers in patients with IDH-mutant gliomas. J Neurooncol 159:261–270. 10.1007/s11060-022-04060-1 35816267 10.1007/s11060-022-04060-1 PMC10183250

[40] Gao F, Cui Y, Jiang H, Sui D, Wang Y, Jiang Z et al (2016) Circulating tumor cell is a common property of brain glioma and promotes the monitoring system. Oncotarget 7:71330–71340. 10.18632/oncotarget.11114 27517490 10.18632/oncotarget.11114 PMC5342081

[41] García-Romero N, Carrión-Navarro J, Esteban-Rubio S, Lázaro-Ibáñez E, Peris-Celda M, Alonso MM et al (2017) DNA sequences within glioma-derived extracellular vesicles can cross the intact blood-brain barrier and be detected in peripheral blood of patients. Oncotarget 8:1416–1428. 10.18632/oncotarget.13635 27902458 10.18632/oncotarget.13635 PMC5352065

[42] Gidlöf O, van der Brug M, Öhman J, Gilje P, Olde B, Wahlestedt C et al (2013) Platelets activated during myocardial infarction release functional miRNA, which can be taken up by endothelial cells and regulate ICAM1 expression. Blood 121:3908–3917. 10.1182/blood-2012-10-461798 23493781 10.1182/blood-2012-10-461798

[43] Gires O, Pan M, Schinke H, Canis M, Baeuerle PA (2020) Expression and function of epithelial cell adhesion molecule EpCAM: Where are we after 40 years? Cancer Metastasis Rev 39:969–987. 10.1007/s10555-020-09898-3 32507912 10.1007/s10555-020-09898-3 PMC7497325

[44] Greuter L, Frank N, Guzman R, Soleman J (2022) The clinical applications of liquid biopsies in pediatric brain tumors: a systematic literature review. Cancers (Basel). 10.3390/cancers14112683 35681663 10.3390/cancers14112683 PMC9179879

[45] Hanssen A, Riebensahm C, Mohme M, Joosse SA, Velthaus JL, Berger LA et al (2018) Frequency of circulating tumor cells (CTC) in patients with brain metastases: implications as a risk assessment marker in oligo-metastatic disease. Cancers (Basel). 10.3390/cancers10120527 30572662 10.3390/cancers10120527 PMC6315958

[46] Haranguș A, Berindan-Neagoe I, Todea DA, Șimon I, Șimon M (2019) Noncoding RNAs and liquid biopsy in lung cancer: a literature review. Diagnostics (Basel). 10.3390/diagnostics9040216 31818027 10.3390/diagnostics9040216 PMC6963838

[47] In ‘t Veld SGJG, Arkani M, Post E, Antunes-Ferreira M, D’Ambrosi S, Vessies DCL et al (2022) Detection and localization of early- and late-stage cancers using platelet RNA. Cancer Cell 40:999-1009.e1006. 10.1016/j.ccell.2022.08.006 36055228 10.1016/j.ccell.2022.08.006

[48] Iorgulescu JB, Blewett T, Xiong K, Crnjac A, Liu R et al (2025) Impact of higher cell-free DNA yields on liquid biopsy testing in glioblastoma patients. Clin Chem 71:215–225. 10.1093/clinchem/hvae178 39749509 10.1093/clinchem/hvae178 PMC12363414

[49] Iser F, Hinz F, Hoffmann DC, Grassl N, Güngoör C, Meyer J et al (2024) Cerebrospinal Fluid cfDNA sequencing for classification of central nervous system glioma. Clin Cancer Res 30:2974–2985. 10.1158/1078-0432.Ccr-23-2907 38295147 10.1158/1078-0432.CCR-23-2907 PMC11247324

[50] Jahr S, Hentze H, Englisch S, Hardt D, Fackelmayer FO, Hesch RD (2001) DNA fragments in the blood plasma of cancer patients: quantitations and evidence for their origin from apoptotic and necrotic cells. Cancer Res 61:1659–1665 11245480

[51] Jeppesen DK, Zhang Q, Franklin JL, Coffey RJ (2023) Extracellular vesicles and nanoparticles: emerging complexities. Trends Cell Biol 33:667–681. 10.1016/j.tcb.2023.01.002 36737375 10.1016/j.tcb.2023.01.002 PMC10363204

[52] Jiang L, Yang H, Cheng W, Ni Z, Xiang N (2023) Droplet microfluidics for CTC-based liquid biopsy: a review. Analyst 148:203–221. 10.1039/d2an01747d 36508171 10.1039/d2an01747d

[53] Karp JM, Modrek AS, Ezhilarasan R, Zhang Z-Y, Ding Y, Graciani M et al (2024) Deconvolution of the tumor-educated platelet transcriptome reveals activated platelet and inflammatory cell transcript signatures. JCI Insight. 10.1172/jci.insight.178719 39190500 10.1172/jci.insight.178719 PMC11466191

[54] Kim BG, Jang JH, Kim JW, Shin SH, Jeong BH, Lee K (2022) Clinical utility of plasma cell-free DNA EGFR mutation analysis in treatment-naïve stage IV non-small cell lung cancer patients. J Clin Med. 10.3390/jcm11041144 35207417 10.3390/jcm11041144 PMC8880481

[55] Klement GL, Yip TT, Cassiola F, Kikuchi L, Cervi D, Podust V et al (2009) Platelets actively sequester angiogenesis regulators. Blood 113:2835–2842. 10.1182/blood-2008-06-159541 19036702 10.1182/blood-2008-06-159541 PMC2661866

[56] Klinsing S, Beck J, Weber KJ, Bornemann-Kolatzki K, Dettki M, Urban H et al (2024) Detection of diagnostic somatic copy number alterations from cerebrospinal fluid cell-free DNA in brain tumor patients. Acta Neuropathol Commun 12:177. 10.1186/s40478-024-01887-9 39568088 10.1186/s40478-024-01887-9 PMC11580493

[57] Klotz R, Thomas A, Teng T, Han SM, Iriondo O, Li L et al (2020) Circulating tumor cells exhibit metastatic tropism and reveal brain metastasis drivers. Cancer Discov 10:86–103. 10.1158/2159-8290.Cd-19-0384 31601552 10.1158/2159-8290.CD-19-0384 PMC6954305

[58] Klotz R, Yu M (2022) Insights into brain metastasis: Recent advances in circulating tumor cell research. Cancer Rep (Hoboken) 5:e1239. 10.1002/cnr2.1239 33372393 10.1002/cnr2.1239 PMC9124503

[59] Labelle M, Begum S, Hynes RO (2011) Direct signaling between platelets and cancer cells induces an epithelial-mesenchymal-like transition and promotes metastasis. Cancer Cell 20:576–590. 10.1016/j.ccr.2011.09.009 22094253 10.1016/j.ccr.2011.09.009 PMC3487108

[60] Labussière M, Di Stefano AL, Gleize V, Boisselier B, Giry M, Mangesius S et al (2014) TERT promoter mutations in gliomas, genetic associations and clinico-pathological correlations. Br J Cancer 111:2024–2032. 10.1038/bjc.2014.538 25314060 10.1038/bjc.2014.538 PMC4229642

[61] Laffont B, Corduan A, Plé H, Duchez A-C, Cloutier N, Boilard E et al (2013) Activated platelets can deliver mRNA regulatory Ago2•microRNA complexes to endothelial cells via microparticles. Blood 122:253–261. 10.1182/blood-2013-03-492801 23652806 10.1182/blood-2013-03-492801

[62] Lamba N, Wen PY, Aizer AA (2021) Epidemiology of brain metastases and leptomeningeal disease. Neuro Oncol 23:1447–1456. 10.1093/neuonc/noab101 33908612 10.1093/neuonc/noab101 PMC8408881

[63] Lazar S, Goldfinger LE (2021) Platelets and extracellular vesicles and their cross talk with cancer. Blood 137:3192–3200. 10.1182/blood.2019004119 33940593 10.1182/blood.2019004119 PMC8351904

[64] Lee JH, Menzies AM, Carlino MS, McEvoy AC, Sandhu S, Weppler AM et al (2020) Longitudinal monitoring of ctDNA in patients with melanoma and brain metastases treated with immune checkpoint inhibitors. Clin Cancer Res 26:4064–4071. 10.1158/1078-0432.Ccr-19-3926 32321716 10.1158/1078-0432.CCR-19-3926

[65] Li M, Chen J, Zhang B, Yu J, Wang N, Li D et al (2022) Dynamic monitoring of cerebrospinal fluid circulating tumor DNA to identify unique genetic profiles of brain metastatic tumors and better predict intracranial tumor responses in non-small cell lung cancer patients with brain metastases: a prospective cohort study (GASTO 1028). BMC Med 20:398. 10.1186/s12916-022-02595-8 36372873 10.1186/s12916-022-02595-8 PMC9661744

[66] Li S, Qu Y, Liu L, Zhang X, He Y, Wang C et al (2023) Comparative proteomic profiling of plasma exosomes in lung cancer cases of liver and brain metastasis. Cell Biosci 13:180. 10.1186/s13578-023-01112-5 37770976 10.1186/s13578-023-01112-5 PMC10540327

[67] Liang J, Zhao W, Lu C, Liu D, Li P, Ye X (2020) Next-generation sequencing analysis of ctDNA for the detection of glioma and metastatic brain tumors in adults. Front Neurol 11:544. 10.3389/fneur.2020.00544 32973641 10.3389/fneur.2020.00544 PMC7473301

[68] Lin D, Shen L, Luo M, Zhang K, Li J, Yang Q et al (2021) Circulating tumor cells: biology and clinical significance. Signal Transduct Target Ther 6:404. 10.1038/s41392-021-00817-8 34803167 10.1038/s41392-021-00817-8 PMC8606574

[69] Lin NU, Lee EQ, Aoyama H, Barani IJ, Barboriak DP, Baumert BG et al (2015) Response assessment criteria for brain metastases: proposal from the RANO group. Lancet Oncol 16:e270-278. 10.1016/s1470-2045(15)70057-4 26065612 10.1016/S1470-2045(15)70057-4

[70] Liu X, Song J, Zhang H, Liu X, Zuo F, Zhao Y et al (2023) Immune checkpoint HLA-E:CD94-NKG2A mediates evasion of circulating tumor cells from NK cell surveillance. Cancer Cell 41:272-287.e279. 10.1016/j.ccell.2023.01.001 36706761 10.1016/j.ccell.2023.01.001

[71] Loreth D, Schuette M, Zinke J, Mohme M, Piffko A, Schneegans S et al (2021) CD74 and CD44 expression on CTCs in cancer patients with brain metastasis. Int J Mol Sci. 10.3390/ijms22136993 34209696 10.3390/ijms22136993 PMC8268634

[72] Lu J, Getz G, Miska EA, Alvarez-Saavedra E, Lamb J, Peck D et al (2005) MicroRNA expression profiles classify human cancers. Nature 435:834–838. 10.1038/nature03702 15944708 10.1038/nature03702

[73] Lubotzky A, Zemmour H, Neiman D, Gotkine M, Loyfer N, Piyanzin S et al (2022) Liquid biopsy reveals collateral tissue damage in cancer. JCI Insight. 10.1172/jci.insight.153559 35076021 10.1172/jci.insight.153559 PMC8855834

[74] Ma C, Yang X, Xing W, Yu H, Si T, Guo Z (2020) Detection of circulating tumor DNA from non-small cell lung cancer brain metastasis in cerebrospinal fluid samples. Thorac Cancer 11:588–593. 10.1111/1759-7714.13300 31944608 10.1111/1759-7714.13300 PMC7049513

[75] Macarthur KM, Kao GD, Chandrasekaran S, Alonso-Basanta M, Chapman C, Lustig RA et al (2014) Detection of brain tumor cells in the peripheral blood by a telomerase promoter-based assay. Cancer Res 74:2152–2159. 10.1158/0008-5472.Can-13-0813 24525740 10.1158/0008-5472.CAN-13-0813 PMC4144786

[76] Maire CL, Fuh MM, Kaulich K, Fita KD, Stevic I, Heiland DH et al (2021) Genome-wide methylation profiling of glioblastoma cell-derived extracellular vesicle DNA allows tumor classification. Neuro Oncol 23:1087–1099. 10.1093/neuonc/noab012 33508126 10.1093/neuonc/noab012 PMC8673443

[77] Mayo ZS, Billena C, Suh JH, Lo SS, Chao ST (2024) The dilemma of radiation necrosis from diagnosis to treatment in the management of brain metastases. Neuro Oncol 26:S56–S65. 10.1093/neuonc/noad188 38437665 10.1093/neuonc/noad188 PMC10911797

[78] Meehan K, Vella LJ (2016) The contribution of tumour-derived exosomes to the hallmarks of cancer. Crit Rev Clin Lab Sci 53:121–131. 10.3109/10408363.2015.1092496 26479834 10.3109/10408363.2015.1092496

[79] Meng Y, Pople CB, Suppiah S, Llinas M, Huang Y, Sahgal A et al (2021) MR-guided focused ultrasound liquid biopsy enriches circulating biomarkers in patients with brain tumors. Neuro Oncol 23:1789–1797. 10.1093/neuonc/noab057 33693781 10.1093/neuonc/noab057 PMC8485448

[80] Miller AM, Shah RH, Pentsova EI, Pourmaleki M, Briggs S, Distefano N et al (2019) Tracking tumour evolution in glioma through liquid biopsies of cerebrospinal fluid. Nature 565:654–658. 10.1038/s41586-019-0882-3 30675060 10.1038/s41586-019-0882-3 PMC6457907

[81] Miller JJ, Gonzalez Castro LN, McBrayer S, Weller M, Cloughesy T, Portnow J et al (2022) Isocitrate dehydrogenase (IDH) mutant gliomas: a society for neuro-oncology (SNO) consensus review on diagnosis, management, and future directions. Neuro Oncol 25:4–25. 10.1093/neuonc/noac207 10.1093/neuonc/noac207 PMC9825337 36239925

[82] Miyamoto S, Yagi H, Yotsumoto F, Kawarabayashi T, Mekada E (2008) Heparin-binding epidermal growth factor-like growth factor as a new target molecule for cancer therapy. Adv Exp Med Biol 622:281–295. 10.1007/978-0-387-68969-2_23 18546636 10.1007/978-0-387-68969-2_23

[83] Moding EJ, Nabet BY, Alizadeh AA, Diehn M (2021) Detecting liquid remnants of solid tumors: circulating tumor DNA minimal residual disease. Cancer Discov 11:2968–2986. 10.1158/2159-8290.Cd-21-0634 34785539 10.1158/2159-8290.CD-21-0634 PMC8976700

[84] Morgan AJ, Giannoudis A, Palmieri C (2021) The genomic landscape of breast cancer brain metastases: a systematic review. Lancet Oncol 22:e7–e17. 10.1016/s1470-2045(20)30556-8 33387511 10.1016/S1470-2045(20)30556-8

[85] Morokoff A, Jones J, Nguyen H, Ma C, Lasocki A, Gaillard F et al (2020) Serum microRNA is a biomarker for post-operative monitoring in glioma. J Neurooncol 149:391–400. 10.1007/s11060-020-03566-w 32915353 10.1007/s11060-020-03566-w

[86] Mouliere F, Smith CG, Heider K, Su J, van der Pol Y, Thompson M et al (2021) Fragmentation patterns and personalized sequencing of cell-free DNA in urine and plasma of glioma patients. EMBO Mol Med 13:e12881. 10.15252/emmm.202012881 34291583 10.15252/emmm.202012881 PMC8350897

[87] Müller Bark J, Kulasinghe A, Hartel G, Leo P, Warkiani ME, Jeffree RL (2021) Isolation of circulating tumour cells in patients with glioblastoma using spiral microfluidic technology - a pilot study. Front Oncol 11:681130. 10.3389/fonc.2021.681130 34150645 10.3389/fonc.2021.681130 PMC8210776

[88] Müller C, Holtschmidt J, Auer M, Heitzer E, Lamszus K, Schulte A et al (2014) Hematogenous dissemination of glioblastoma multiforme. Sci Transl Med. 10.1126/scitranslmed.3009095 25080476 10.1126/scitranslmed.3009095

[89] Muralidharan K, Yekula A, Small JL, Rosh ZS, Kang KM, Wang L et al (2021) TERT promoter mutation analysis for blood-based diagnosis and monitoring of gliomas. Clin Cancer Res 27:169–178. 10.1158/1078-0432.Ccr-20-3083 33051308 10.1158/1078-0432.CCR-20-3083 PMC7785705

[90] Naito T, Tanaka F, Ono A, Yoneda K, Takahashi T, Murakami H et al (2012) Prognostic impact of circulating tumor cells in patients with small cell lung cancer. J Thorac Oncol 7:512–519. 10.1097/JTO.0b013e31823f125d 22258473 10.1097/JTO.0b013e31823f125d

[91] Nassa G, Giurato G, Cimmino G, Rizzo F, Ravo M, Salvati A et al (2018) Splicing of platelet resident pre-mRNAs upon activation by physiological stimuli results in functionally relevant proteome modifications. Sci Rep 8:498. 10.1038/s41598-017-18985-5 29323256 10.1038/s41598-017-18985-5 PMC5765118

[92] Nassiri F, Chakravarthy A, Feng S, Shen SY, Nejad R, Zuccato JA et al (2020) Detection and discrimination of intracranial tumors using plasma cell-free DNA methylomes. Nat Med 26:1044–1047. 10.1038/s41591-020-0932-2 32572265 10.1038/s41591-020-0932-2 PMC8500275

[93] Nayak L, Lee EQ, Wen PY (2012) Epidemiology of brain metastases. Curr Oncol Rep 14:48–54. 10.1007/s11912-011-0203-y 22012633 10.1007/s11912-011-0203-y

[94] Nilsson RJ, Balaj L, Hulleman E, van Rijn S, Pegtel DM, Walraven M et al (2011) Blood platelets contain tumor-derived RNA biomarkers. Blood 118:3680–3683. 10.1182/blood-2011-03-344408 21832279 10.1182/blood-2011-03-344408 PMC7224637

[95] Nørøxe DS, Østrup O, Yde CW, Ahlborn LB, Nielsen FC, Michaelsen SR et al (2019) Cell-free DNA in newly diagnosed patients with glioblastoma - a clinical prospective feasibility study. Oncotarget 10:4397–4406. 10.18632/oncotarget.27030 31320993 10.18632/oncotarget.27030 PMC6633897

[96] Ostrom QT, Price M, Neff C, Cioffi G, Waite KA, Kruchko C et al (2023) CBTRUS statistical report: primary brain and other central nervous system tumors diagnosed in the United States in 2016–2020. Neuro Oncol. 10.1093/neuonc/noad149 37793125 10.1093/neuonc/noad149 PMC10550277

[97] Pangeni RP, Olivaries I, Huen D, Buzatto VC, Dawson TP, Ashton KM et al (2022) Genome-wide methylation analyses identifies Non-coding RNA genes dysregulated in breast tumours that metastasise to the brain. Sci Rep 12:1102. 10.1038/s41598-022-05050-z 35058523 10.1038/s41598-022-05050-z PMC8776809

[98] Pedrosa R, Mustafa DA, Soffietti R, Kros JM (2018) Breast cancer brain metastasis: molecular mechanisms and directions for treatment. Neuro Oncol 20:1439–1449. 10.1093/neuonc/noy044 29566179 10.1093/neuonc/noy044 PMC6176797

[99] Piccioni DE, Achrol AS, Kiedrowski LA, Banks KC, Boucher N, Barkhoudarian G et al (2019) Analysis of cell-free circulating tumor DNA in 419 patients with glioblastoma and other primary brain tumors. CNS Oncol. 10.2217/cns-2018-0015 30855176 10.2217/cns-2018-0015 PMC6713031

[100] Pierga JY, Bidard FC, Cropet C, Tresca P, Dalenc F, Romieu G et al (2013) Circulating tumor cells and brain metastasis outcome in patients with HER2-positive breast cancer: the LANDSCAPE trial. Ann Oncol 24:2999–3004. 10.1093/annonc/mdt348 24013510 10.1093/annonc/mdt348

[101] Placke T, Örgel M, Schaller M, Jung G, Rammensee HG, Kopp HG et al (2012) Platelet-derived MHC class I confers a pseudonormal phenotype to cancer cells that subverts the antitumor reactivity of natural killer immune cells. Cancer Res 72:440–448. 10.1158/0008-5472.Can-11-1872 22127925 10.1158/0008-5472.CAN-11-1872

[102] Qi Y, Xu Y, Yan T, Xiong W, Zhu X, Sun Q et al (2024) Use of circulating tumor cells and microemboli to predict diagnosis and prognosis in diffuse glioma. J Neurosurg 141:673–683. 10.3171/2024.1.JNS232020 38608304 10.3171/2024.1.JNS232020

[103] Rath B, Klameth L, Plangger A, Hochmair M, Ulsperger E, Huk I et al (2019) Expression of Proteolytic Enzymes by Small Cell Lung Cancer Circulating Tumor Cell Lines. Cancers (Basel). 10.3390/cancers11010114 30669448 10.3390/cancers11010114 PMC6357007

[104] Rehman AU, Khan P, Maurya SK, Siddiqui JA, Santamaria-Barria JA, Batra SK et al (2022) Liquid biopsies to occult brain metastasis. Mol Cancer 21:113. 10.1186/s12943-022-01577-x 35538484 10.1186/s12943-022-01577-x PMC9088117

[105] Ricklefs FL, Wollmann K, Salviano-Silva A, Drexler R, Maire CL, Kaul MG et al (2024) Circulating extracellular vesicles as biomarker for diagnosis, prognosis, and monitoring in glioblastoma patients. Neuro Oncol 26:1280–1291. 10.1093/neuonc/noae068 38567448 10.1093/neuonc/noae068 PMC11226867

[106] Riebensahm C, Joosse SA, Mohme M, Hanssen A, Matschke J, Goy Y et al (2019) Clonality of circulating tumor cells in breast cancer brain metastasis patients. Breast Cancer Res 21:101. 10.1186/s13058-019-1184-2 31481116 10.1186/s13058-019-1184-2 PMC6720990

[107] Risitano A, Beaulieu LM, Vitseva O, Freedman JE (2012) Platelets and platelet-like particles mediate intercellular RNA transfer. Blood 119:6288–6295. 10.1182/blood-2011-12-396440 22596260 10.1182/blood-2011-12-396440 PMC3383198

[108] Riviere-Cazaux C, Dong X, Mo W, Dai C, Carlstrom LP, Munoz-Casabella A et al (2024) Longitudinal glioma monitoring via cerebrospinal fluid cell-free DNA: one patient at a time. medRxiv. 10.1101/2024.02.21.24303164 39715486 10.1158/1078-0432.CCR-24-1814 PMC11873801

[109] Riviere-Cazaux C, Lacey JM, Carlstrom LP, Laxen WJ, Munoz-Casabella A, Hoplin MD et al (2023) Cerebrospinal fluid 2-hydroxyglutarate as a monitoring biomarker for IDH-mutant gliomas. Neuro-Oncol Adv. 10.1093/noajnl/vdad061 10.1093/noajnl/vdad061 PMC10259246 37313502

[110] Rodrigues G, Hoshino A, Kenific CM, Matei IR, Steiner L, Freitas D et al (2019) Tumour exosomal CEMIP protein promotes cancer cell colonization in brain metastasis. Nat Cell Biol 21:1403–1412. 10.1038/s41556-019-0404-4 31685984 10.1038/s41556-019-0404-4 PMC7354005

[111] Ruan X, Yan W, Cao M, Daza RAM, Fong MY, Yang K et al (2024) Breast cancer cell-secreted miR-199b-5p hijacks neurometabolic coupling to promote brain metastasis. Nat Commun 15:4549. 10.1038/s41467-024-48740-0 38811525 10.1038/s41467-024-48740-0 PMC11137082

[112] Sabedot TS, Malta TM, Snyder J, Nelson K, Wells M, deCarvalho AC et al (2021) A serum-based DNA methylation assay provides accurate detection of glioma. Neuro Oncol 23:1494–1508. 10.1093/neuonc/noab023 33560371 10.1093/neuonc/noab023 PMC8408843

[113] Sato J, Shimomura A, Kawauchi J, Matsuzaki J, Yamamoto Y, Takizawa S et al (2019) Brain metastasis-related microRNAs in patients with advanced breast cancer. PLoS ONE 14:e0221538. 10.1371/journal.pone.0221538 31603918 10.1371/journal.pone.0221538 PMC6788729

[114] Schwaederle M, Husain H, Fanta PT, Piccioni DE, Kesari S, Schwab RB et al (2016) Detection rate of actionable mutations in diverse cancers using a biopsy-free (blood) circulating tumor cell DNA assay. Oncotarget 7:9707–9717. 10.18632/oncotarget.7110 26848768 10.18632/oncotarget.7110 PMC4891078

[115] Seremet T, Jansen Y, Planken S, Njimi H, Delaunoy M, El Housni H et al (2019) Undetectable circulating tumor DNA (ctDNA) levels correlate with favorable outcome in metastatic melanoma patients treated with anti-PD1 therapy. J Transl Med 17:303. 10.1186/s12967-019-2051-8 31488153 10.1186/s12967-019-2051-8 PMC6727487

[116] Sheng Z, Bu C, Mei J, Xu S, Zhang Z, Guo G et al (2023) Tracking tumor evolution during the first-line treatment in brain glioma via serial profiling of cell-free tumor DNA from tumor in situ fluid. Front Oncol 13:1238607. 10.3389/fonc.2023.1238607 37920153 10.3389/fonc.2023.1238607 PMC10619896

[117] Short SC, Noushmehr H (2022) Unmet need for liquid biomarkers and the brain-liquid biopsy consortium. Neurooncol Adv. 10.1093/noajnl/vdac020 36380869 10.1093/noajnl/vdac020 PMC9650471

[118] Skog J, Würdinger T, van Rijn S, Meijer DH, Gainche L, Curry WT et al (2008) Glioblastoma microvesicles transport RNA and proteins that promote tumour growth and provide diagnostic biomarkers. Nat Cell Biol 10:1470–1476. 10.1038/ncb1800 19011622 10.1038/ncb1800 PMC3423894

[119] Sol N, In ‘t Veld S, Vancura A, Tjerkstra M, Leurs C, Rustenburg F et al (2020) Tumor-educated platelet RNA for the detection and (pseudo)progression monitoring of glioblastoma. Cell Rep Med 1:100101. 10.1016/j.xcrm.2020.100101 33103128 10.1016/j.xcrm.2020.100101 PMC7576690

[120] Sol N, Kooi EJ, Pagès-Gallego M, Brandsma D, Bugiani M, de Ridder J et al (2024) Glioblastoma, IDH-wildtype with primarily leptomeningeal localization diagnosed by nanopore sequencing of cell-free DNA from cerebrospinal fluid. Acta Neuropathol 148:35. 10.1007/s00401-024-02792-0 39225924 10.1007/s00401-024-02792-0 PMC11371860

[121] Soler DC, Kerstetter-Fogle A, Elder T, Raghavan A, Barnholtz-Sloan JS, Cooper KD et al (2020) A liquid biopsy to assess brain tumor recurrence: presence of circulating Mo-MDSC and CD14+ VNN2+ myeloid cells as biomarkers that distinguish brain metastasis from radiation necrosis following stereotactic radiosurgery. Neurosurgery 88:E67-e72. 10.1093/neuros/nyaa334 32823285 10.1093/neuros/nyaa334 PMC8861507

[122] Sparano J, O’Neill A, Alpaugh K, Wolff AC, Northfelt DW, Dang CT et al (2018) Association of circulating tumor cells with late recurrence of estrogen receptor-positive breast cancer: a secondary analysis of a randomized clinical trial. JAMA Oncol 4:1700–1706. 10.1001/jamaoncol.2018.2574 30054636 10.1001/jamaoncol.2018.2574 PMC6385891

[123] Sullivan JP, Nahed BV, Madden MW, Oliveira SM, Springer S, Bhere D et al (2014) Brain tumor cells in circulation are enriched for mesenchymal gene expression. Cancer Discov 4:1299–1309. 10.1158/2159-8290.Cd-14-0471 25139148 10.1158/2159-8290.CD-14-0471 PMC4221467

[124] Tripathy A, John V, Wadden J, Kong S, Sharba S, Koschmann C (2022) Liquid biopsy in pediatric brain tumors. Front Genet 13:1114762. 10.3389/fgene.2022.1114762 36685825 10.3389/fgene.2022.1114762 PMC9853427

[125] van den Bent MJ, Geurts M, French PJ, Smits M, Capper D, Bromberg JEC et al (2023) Primary brain tumours in adults. The Lancet 402:1564–1579. 10.1016/S0140-6736(23)01054-1 10.1016/S0140-6736(23)01054-1 37738997

[126] Vishnoi M, Peddibhotla S, Yin W, Ts A, George GC, Hong DS et al (2015) The isolation and characterization of CTC subsets related to breast cancer dormancy. Sci Rep 5:17533. 10.1038/srep17533 26631983 10.1038/srep17533 PMC4668355

[127] Wan JCM, Massie C, Garcia-Corbacho J, Mouliere F, Brenton JD, Caldas C et al (2017) Liquid biopsies come of age: towards implementation of circulating tumour DNA. Nat Rev Cancer 17:223–238. 10.1038/nrc.2017.7 28233803 10.1038/nrc.2017.7

[128] Wang S, Zhang K, Tan S, Xin J, Yuan Q, Xu H et al (2021) Circular RNAs in body fluids as cancer biomarkers: the new frontier of liquid biopsies. Mol Cancer 20:13. 10.1186/s12943-020-01298-z 33430880 10.1186/s12943-020-01298-z PMC7798340

[129] Wei C, Zhang R, Cai Q, Gao X, Tong F, Dong J et al (2019) MicroRNA-330-3p promotes brain metastasis and epithelial-mesenchymal transition via GRIA3 in non-small cell lung cancer. Aging (Albany NY) 11:6734–6761. 10.18632/aging.102201 31498117 10.18632/aging.102201 PMC6756898

[130] Wei L, Wang G, Yang C, Zhang Y, Chen Y, Zhong C et al (2021) MicroRNA-550a-3-5p controls the brain metastasis of lung cancer by directly targeting YAP1. Cancer Cell Int 21:491. 10.1186/s12935-021-02197-z 34530822 10.1186/s12935-021-02197-z PMC8444378

[131] Weller M, van den Bent M, Preusser M, Le Rhun E, Tonn JC, Minniti G et al (2021) EANO guidelines on the diagnosis and treatment of diffuse gliomas of adulthood. Nat Rev Clin Oncol 18:170–186. 10.1038/s41571-020-00447-z 33293629 10.1038/s41571-020-00447-z PMC7904519

[132] Welsh JA, Goberdhan DCI, O’Driscoll L, Buzas EI, Blenkiron C, Bussolati B et al (2024) Minimal information for studies of extracellular vesicles (MISEV2023): From basic to advanced approaches. J Extracell Ves 13:e12404. 10.1002/jev2.12404 10.1002/jev2.12404 PMC10850029 38326288

[133] Wen PY, van den Bent M, Youssef G, Cloughesy TF, Ellingson BM, Weller M et al (2023) RANO 2.0: update to the response assessment in neuro-oncology criteria for high- and low-grade gliomas in adults. J Clin Oncol 41:5187–5199. 10.1200/jco.23.01059 37774317 10.1200/JCO.23.01059 PMC10860967

[134] Wen PY, Weller M, Lee EQ, Alexander BM, Barnholtz-Sloan JS, Barthel FP et al (2020) Glioblastoma in adults: a Society for Neuro-Oncology (SNO) and European Society of Neuro-Oncology (EANO) consensus review on current management and future directions. Neuro Oncol 22:1073–1113. 10.1093/neuonc/noaa106 32328653 10.1093/neuonc/noaa106 PMC7594557

[135] Wu F, McCuaig RD, Sutton CR, Tan AHY, Jeelall Y, Bean EG et al (2019) Nuclear-biased DUSP6 expression is associated with cancer spreading including brain metastasis in triple-negative breast cancer. Int J Mol Sci. 10.3390/ijms20123080 31238530 10.3390/ijms20123080 PMC6627630

[136] Wu J, Liu Z, Huang T, Wang Y, Song MM, Song T et al (2023) Cerebrospinal fluid circulating tumor DNA depicts profiling of brain metastasis in NSCLC. Mol Oncol 17:810–824. 10.1002/1878-0261.13357 36495130 10.1002/1878-0261.13357 PMC10158766

[137] Yuan J, Xu L, Chien CY, Yang Y, Yue Y, Fadera S et al (2023) First-in-human prospective trial of sonobiopsy in high-grade glioma patients using neuronavigation-guided focused ultrasound. NPJ Precis Oncol 7:92. 10.1038/s41698-023-00448-y 37717084 10.1038/s41698-023-00448-y PMC10505140

[138] Zhan Q, Liu B, Situ X, Luo Y, Fu T, Wang Y et al (2023) New insights into the correlations between circulating tumor cells and target organ metastasis. Signal Transduct Target Ther 8:465. 10.1038/s41392-023-01725-9 38129401 10.1038/s41392-023-01725-9 PMC10739776

[139] Zhang L, Ridgway LD, Wetzel MD, Ngo J, Yin W, Kumar D et al (2013) The identification and characterization of breast cancer CTCs competent for brain metastasis. Sci Transl Med 5:180ra148. 10.1126/scitranslmed.3005109 10.1126/scitranslmed.3005109 PMC3863909 23576814

[140] Zhang W, Bao L, Yang S, Qian Z, Dong M, Yin L et al (2016) Tumor-selective replication herpes simplex virus-based technology significantly improves clinical detection and prognostication of viable circulating tumor cells. Oncotarget 7:39768–39783. 10.18632/oncotarget.9465 27206795 10.18632/oncotarget.9465 PMC5129969

[141] Zhang W, Qin T, Yang Z, Yin L, Zhao C, Feng L et al (2021) Telomerase-positive circulating tumor cells are associated with poor prognosis via a neutrophil-mediated inflammatory immune environment in glioma. BMC Med 19:277. 10.1186/s12916-021-02138-7 34763698 10.1186/s12916-021-02138-7 PMC8588721

[142] Zhi F, Shao N, Wang R, Deng D, Xue L, Wang Q et al (2014) Identification of 9 serum microRNAs as potential noninvasive biomarkers of human astrocytoma. Neuro Oncol 17:383–391. 10.1093/neuonc/nou169 25140035 10.1093/neuonc/nou169 PMC4483096

[143] Zill OA, Banks KC, Fairclough SR, Mortimer SA, Vowles JV, Mokhtari R et al (2018) The landscape of actionable genomic alterations in cell-free circulating tumor DNA from 21,807 advanced cancer patients. Clin Cancer Res 24:3528–3538. 10.1158/1078-0432.Ccr-17-3837 29776953 10.1158/1078-0432.CCR-17-3837

[144] Zuccato JA, Mamatjan Y, Nassiri F, Ajisebutu A, Liu JC, Muazzam A et al (2024) Prediction of brain metastasis development with DNA methylation signatures. Nature Med. 10.1038/s41591-024-03286-y 39379704 10.1038/s41591-024-03286-y PMC11750707

